# Where to Dig for Fossils: Combining Climate-Envelope, Taphonomy and Discovery Models

**DOI:** 10.1371/journal.pone.0151090

**Published:** 2016-03-30

**Authors:** Sebastián Block, Frédérik Saltré, Marta Rodríguez-Rey, Damien A. Fordham, Ingmar Unkel, Corey J. A. Bradshaw

**Affiliations:** 1 The Environment Institute and School of Biological Sciences, The University of Adelaide, Adelaide, South Australia, Australia; 2 Institute for Ecosystem Research, Kiel University, Kiel, Schleswig-Holstein, Germany; Penn State University, UNITED STATES

## Abstract

Fossils represent invaluable data to reconstruct the past history of life, yet fossil-rich sites are often rare and difficult to find. The traditional fossil-hunting approach focuses on small areas and has not yet taken advantage of modelling techniques commonly used in ecology to account for an organism’s past distributions. We propose a new method to assist finding fossils at continental scales based on modelling the past distribution of species, the geological suitability of fossil preservation and the likelihood of fossil discovery in the field, and apply it to several genera of Australian megafauna that went extinct in the Late Quaternary. Our models predicted higher fossil potentials for independent sites than for randomly selected locations (mean Kolmogorov-Smirnov statistic = 0.66). We demonstrate the utility of accounting for the distribution history of fossil taxa when trying to find the most suitable areas to look for fossils. For some genera, the probability of finding fossils based on simple climate-envelope models was higher than the probability based on models incorporating current conditions associated with fossil preservation and discovery as predictors. However, combining the outputs from climate-envelope, preservation, and discovery models resulted in the most accurate predictions of potential fossil sites at a continental scale. We proposed potential areas to discover new fossils of *Diprotodon*, *Zygomaturus*, *Protemnodon*, *Thylacoleo*, and *Genyornis*, and provide guidelines on how to apply our approach to assist fossil hunting in other continents and geological settings.

## Introduction

About 99% of all the species that have evolved on Earth are extinct [1], and fossils are the main source of information we have to describe them [2,3]. Moreover, fossils are also valuable for understanding how current ecological communities might respond to environmental changes [4–6]. However, fossils of many species are exceedingly rare because their formation and persistence depend on a series of unlikely events and conditions. Fossil formation is usually the result of an organism’s remains being rapidly buried in sediments and preserved (e.g., by mineralisation or compression). Subsequent exposure by erosion or crust movement can promote fossil discovery, but intense erosion can also destroy the fossil itself [3].

The standard approach to find fossils is by prospecting at excavation sites and surrounding areas that are already known for their fossil assemblages [3]. While these methods have led to many successful fossil discoveries, most novel finds occur in sites previously unknown for their fossil assemblages; identifying such new sites over potentially vast areas is technically challenging using the traditional approach. More recently, potential fossil sites have been identified using remote sensing and machine-learning algorithms [7–11]. Machine-learning algorithms can classify the pixels of a satellite image to identify the spectral properties of fossil sites and infer potential site locations at fine scales (e.g., a Landsat 7 image has a pixel resolution of 30 m) [10]. Despite successful applications, these methods are limited to small areas (e.g., a single catchment or geological formation) and neglect the climatic conditions that constrained species’ distributions, and how these changed through time. Accounting for variations in distributional ranges could improve predictions of new fossil locations and allow fossil searches to be targeted to species of particular interest.

We developed a new modelling approach for species-specific fossil hunting at continental scales, taking into account species’ geographical range limits and their variation through time. We coupled three statistical models that spatially predict the suitability for (*i*) species occurrence over the last 120 ka (ka = 10^3^ years) given palaeo-climate conditions (mean annual temperature and precipitation), (*ii*) fossil preservation given geological constraints, and (*iii*) the suitability for fossil exposure given present-day environmental conditions. As an example, we applied the method to find new potential fossil areas for five genera of Late Pleistocene megafauna in Australia: *Diprotodon*, *Zygomaturus*, *Protemnodon*, *Thylacoleo*, and *Genyornis*. We showed that averaging the ranking of these three suitability values can help identify areas in which to focus future fossil-hunting, and that accounting for spatio-temporal variation in geographic range improves predictions of new fossil sites for some genera.

## Methods

**Fig 1:**
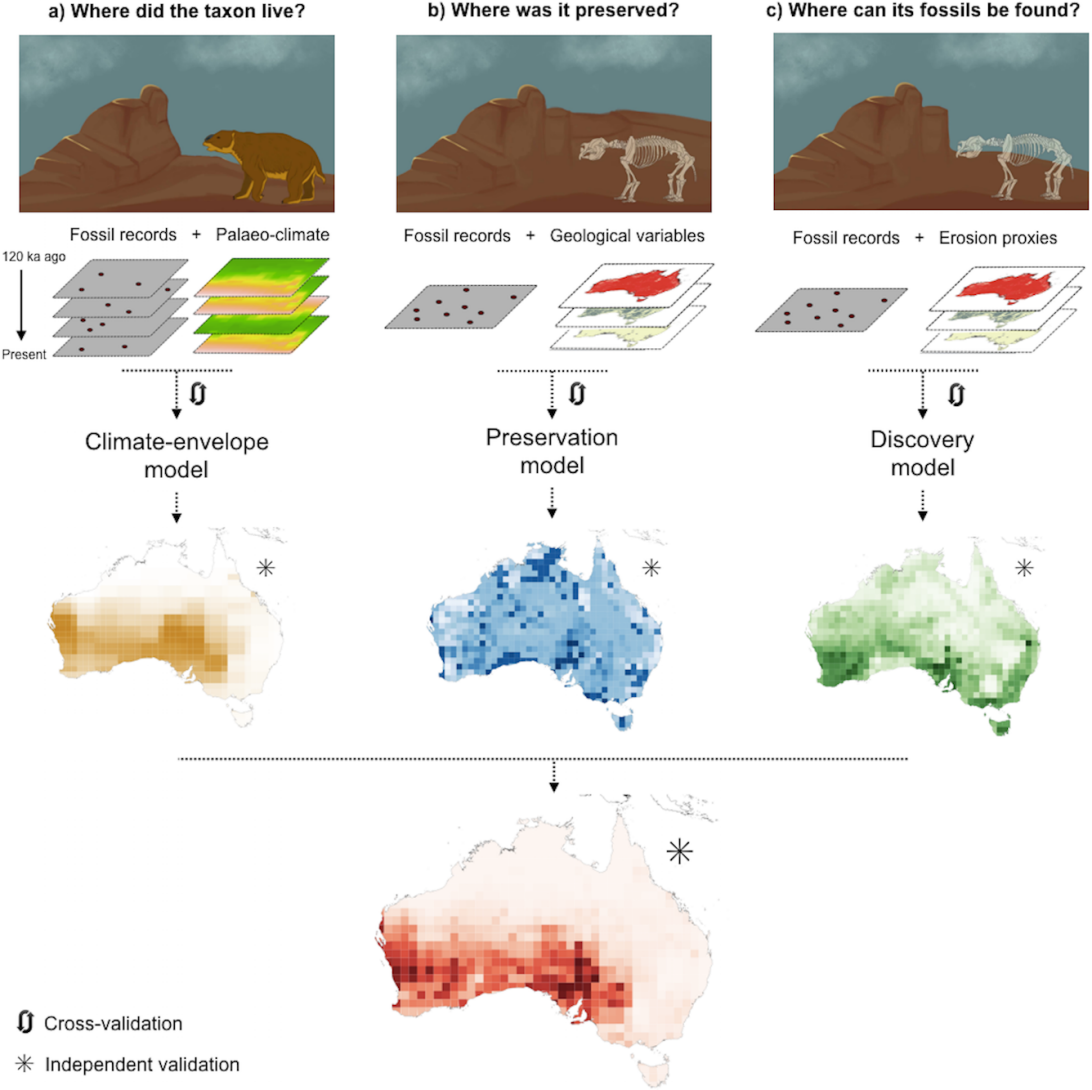
Approach to identify potential fossil areas with combined models. For a given taxon, the areas with greatest potential to yield new fossils (red map) are those where the species used to live (brown map), where its fossils could be preserved (blue map), and where it is now possible to find its fossils (green map). (a) We used palaeo-climate data and fossil records with reliable ages to model the climate envelope of different genera of the Australian megafauna, geological variables to model the suitability for fossil preservation (b) and erosion proxies to model the suitability for fossil discovery (c). The average of the suitability rankings predicted by the climate-envelope, preservation, and discovery models can be used as an indicator of the potential of an area to yield new fossils of a given taxon. We cross-validated each model and used an independent subset of the data to validate the final predictions of each model and their combination.

We modelled the likelihood of finding fossils of a given taxon (i.e., genus or species) in Australia at a grid cell resolution of 1 × 1°. In each grid cell, we assumed that the likelihood of finding fossils of a given taxon depends on the following three criteria: (*i*) suitable climatic conditions over the last 120 ka for the taxon to live (Fig 1A), (*ii*) suitable geological conditions for fossil preservation (Fig 1B) and (*iii*) suitable present-day environmental conditions for fossil discovery (Fig 1C). We built a separate statistical model for each criterion. We only had presence-background data (i.e, we lacked reliable records of fossil absences), so we interpret the model outputs as rankings of each grid cell’s suitability to meet each criterion, rather than true probabilities of fossil occurrence [12]. Thus, we ranked each model’s raw outputs and calculated the average of the three rankings to obtain a final value of a grid cell’s potential to yield new fossils (i.e., the output of each model was equally weighted in the combination).

We applied our approach to identify new fossils areas for five extinct genera of Australian megafauna: *Diprotodon*, the largest marsupial that ever existed; *Zygomaturus*, sometimes called the ‘marsupial rhino’; *Protemnodon*, the giant wallaby; *Thylacoleo*, the marsupial lion; and *Genyornis*, the mihirung, an ostrich-sized, flightless bird. We selected these genera because we had sufficient fossil records (*n* > 10) with good spatio-temporal coverage (i.e., present in at least three grid cells). We extracted fossil records from the *FosSahul* database (Australian Ecological Knowledge and Observation System Data Portal, doi: 10.4227/05/564E6209C4FE8) [13], in which the quality of each fossil’s age was rated and assigned to one of four categories (A*, A, B, or C, in decreasing quality). The quality rating is based on (1) the reliability of the dating and pretreatment protocols and (2) the association between the target fossil and the dated materials [14]. There are different criteria for different dating techniques. For example, reliable radiocarbon ages can be obtained from well-preserved collagen pretreated with ultrafiltration, XAD-2, or ninhydrin protocols to remove possible contaminants [15]; reliable uranium-series ages can be obtained from materials that act as either chemically closed systems or as open systems when combined with modelling of uranium-migration processes. When remains of the target species are not directly dated, ages are only reliable if they come from contexts with stratigraphic integrity [14]. The fossils of the five genera had reliable ages (categories A* and A) ranging from 120 to about 40 ka ago and had a similar spatial distribution, with the exception of *Genyornis* (S1 Fig). We calibrated radiocarbon ages using the Southern Hemisphere Calibration curve (SHCal13) from the OxCal radiocarbon calibration tool Version 4.2 [16].

### Palaeo-climate suitability for taxon occurrence

Besides rare translocation events (movement of fossils from the original place of an organism’s death) [17], fossils of a given taxon are only found in places where the taxon once lived. By pairing fossil records with palaeo-climatic conditions that coincide with the approximate time at which the organism was alive (fossil age) we can estimate climatic suitability (i.e., a taxon’s climate envelope) across space and time [18,19].

We used absolute values of mean annual temperature and total annual precipitation from the Hadley Centre climate model (HadCM3) simulations for the last 120 ka, available at a spatial resolution of 1° and at 1 ka time slices between 0 and 22 ka ago; 2 ka time slices between 22 and 80 ka ago; and 4 ka time slices beyond 80 ka ago [20]. These climate layers have been used previously to estimate timing of megafauna extinction in Australia [21]. Although species’ ranges are likely constrained by a diverse suite of environmental conditions [22], we followed the approach of previous studies by assuming that annual temperature and precipitation are reasonable predictors of past ranges of megafauna [19]. As a response variable, we used fossil presences of the five Australian megafauna genera (we could not use species-level information because of the small size of our samples). To account for the uncertainty of fossil ages we only used fossils with reliable ages (A* and A categories in [14])(S1 Fig) and selected the palaeo-climate slices nearest in time to the mean fossil age (± both 1 and 2 standard deviations), and calculated the Gaussian-weighted average of climate values of these time slices (i.e., the closer a time slice to the mean fossil age, the more it influenced the calculation of the average climate values).

In addition to presence data, most climate-envelope models require data of the climatic conditions in which the species has not been recorded (background data) or is assumed to be absent (pseudo-absence data) [12]. We selected pseudo-absences from fossil sites where the taxon of interest was absent because the accuracy of climate-envelope model predictions can be improved by selecting pseudo-absences with the same biases as are inherent (but not necessarily known) in the presence dataset [23]. However, the observation that a taxon is absent from a fossil site does not necessarily mean that it never occurred in that area [24]. To reduce the risk of including false absences, we only selected pseudo-absences from outside the climatic envelope of the genus (climates with either temperature or precipitation values < the 5^th^ or > the 95^th^ percentiles of the climate values of the presence data) [25]. We selected ten times more pseudo-absences than presences for all modelled genera except *Genyornis*, where we selected all pseudo-absences that met the criteria due to a lack of fossil sites (1312 pseudo-absences for 148 presences).

We modelled climate envelopes using three different methods: Bioclim [26], MaxEnt [27] and generalised linear models [28] (see details in S1 Appendix) because predictions can be sensitive to the method used to estimate climate suitability (e.g., MaxEnt predictions tend to be less sensitive to sample sizes) [29–31]. We evaluated each modelling method using (*i*) a ‘spatio-temporal’ validation and (*ii*) a ‘temporal-only’ validation. In the spatio-temporal validation, we pooled all the data and ran a five-fold cross-validation, so that data used for training and testing came from different points in time and space. In each round of the temporal-only cross-validation, we excluded the data of one time-slice for model training and used it for validation. We had as many rounds as time slices with fossils of the genus so that fossils from each time slice were used for model training and validation. We assessed predictive accuracy using the true skill statistic, which is the sum of the sensitivity (the proportion of presences predicted correctly) and specificity (the proportion of absences predicted correctly) minus one [32]. We projected climate suitability in each grid cell for each time slice in which the taxon was still alive (i.e., the time-slice with the youngest fossil record and all the previous ones) by weighting the projections from each model by its true skill statistic [33]. Lastly, we averaged climate suitability in each grid cell across all time slices. We used the R package *dismo* to generate all climate-envelope models [34,35]. The code is available at https://github.com/seblun/Fossil-hunting-models.

### Geological suitability for fossil preservation

We used logistic regression to model the suitability of fossil preservation in each grid cell as a function of three geological constraints: suitable-rock cover, lake cover, and cave presence. We assumed that these variables are relevant predictors of fossil preservation because Australian megafauna fossils are almost always found in sedimentary rocks and regoliths [3], and caves and lakes (the richest localities of Late Quaternary fossils in Australia [13]) work as pit traps leading to fossil accumulation and provide adequate conditions for their preservation [17,36]. We also assumed that geological conditions did not change over the time scale under consideration (last 120 ka) and that certain environments are more conducive to fossilisation than others [37].

We used freely available datasets to extract the geological predictors and calculated their values in each grid cell. We estimated sedimentary rocks and regoliths in each grid cell using the surface geology of Australia 1:10^6^ scale dataset [38] processed with QGIS [39]. We extracted geospatial data of lakes and caves in Australia from the GEODATA TOPO 250k Series 3 topographic database [40] and quantified the area of lakes and the presence of caves (as a binary variable) in each grid cell.

For the models of suitability for fossil preservation and discovery, we used all fossil records disregarding their taxonomic identity and age quality, because the mere presence of a fossil at a site demonstrates that fossils can be preserved and discovered there (i.e., irrespective of species identity and the reliability of the fossil age). The response variable was the presence or absence of fossils at the grid cell level (1 × 1°) rather than fossil density to avoid any bias due to the spatial aggregation arising from prospecting (i.e., many fossil sites in the same grid cell can result in a biased measure of fossil density) [10]. Of the 849 grid cells encompassing Australia, 103 had fossils (S2 Fig).

### Suitability of the present-day environment for fossil discovery

We modelled the suitability for fossil discovery as a function of erosion proxies: mean slope, rain intensity and bare soil cover in each grid cell. Our key assumption was that erosion can expose fossils, and thus improve the chances of finding them while prospecting. We created a slope map of Australia from a digital elevation model [41] with the Raster Terrain Analysis plugin of QGIS [39], and calculated the rain intensity (as a proxy of its erosive power) by dividing the mean annual precipitation [42] by the mean annual days of rain [43] (data from the Australian Government’s Bureau of Meteorology – www.bom.gov.au). Finally, we calculated the bare soil cover in each grid cell using a map of Australia’s vegetation in the mid-1980s [44] that shows areas with no vegetation (bare soil).

Fossil presence data are often spatially biased because sampling is concentrated in the areas most accessible to humans. To account for this potential bias, we investigated the relative role of slope, rain intensity, and bare soil cover in predicting fossil presence without the confounding effect of site accessibility. We modelled the sampling probability in every grid cell and used its reciprocal to weight the observations in the fossil-discovery model, so that grid cells with high probabilities of being sampled (and where fossil prospecting has arguably been more intense) were less important in the model [45]. As proxies of accessibility (and thus of sampling effort), we calculated the human population and road density per grid cell, and the distance of each grid cell’s centroid to the centroid of large and medium cities (> 1 million and > 50 thousand people, respectively) [46]. Using fossil presence as the response variable, we fitted 16 logistic regressions with all combinations of these four explanatory variables and ranked them based on their Bayesian information criteria [47]. This resulted in incorporating only human population density and distance to large cities as explanatory variables in the most parsimonious model (S1 Table). Using the reciprocal of this model’s output to weight the observations in the fossil discovery model, we reduced the importance of (potentially) heavily sampled grid cells, so that the estimated coefficients for slope, rain intensity, and bare soil cover represented the role of these variables as predictors of fossil presence when accounting for sampling bias. We then used these coefficients to predict the suitability of fossil discovery without using sampling-bias weights because there was no intrinsic reason why the suitability of fossil discovery should change with the accessibility to the site (i.e., sampling-bias weights are useful to elucidate the role of predictors of fossil-discovery suitability, but not to make the predictions).

### Validation and analyses

For each of the three statistical models, we did a five-fold cross-validation and in each validation round we calculated the true skill statistic and area under the receiver operating characteristic curve. In addition, we trained the climate-envelope, preservation, and discovery models excluding grid cells with unreliably dated fossils of the five megafauna genera, and using them to test the skill of model predictions (and their combinations) in two ways. The first test validated the continuous output of the models and the second was based on binary (suitable or unsuitable) output.

In the first validation, we used a Kolmogorov-Smirnov test to compare the cumulative distribution of the suitability predictions at independent validation sites against randomly selected grid cells. The Kolmogorov-Smirnov statistic ranges from 0 to 1 and denotes the maximum difference between the two cumulative distributions being compared (i.e., 1 means that all suitabilities predicted in grid cells with fossils are larger than the suitabilities predicted in randomly selected grid cells) [48].

In the second validation, we compared the probabilities of finding grid cells with fossils in ‘suitable’ areas identified by the models versus the probability of finding them at random. In particular, we compared probabilities in (*i*) the overlap of areas suitable for preservation and discovery (the focus of previous modelling attempts to find fossils)[49], (*ii*) areas of suitable palaeo-climate, and (*iii*) the overlap of the three areas (palaeo-climate, preservation, and discovery). We used thresholds that maximised the true skill statistic to transform the continuous output of the models into binary predictions. By using a threshold that maximised the true skill statistic, we obtained areas that included as many presences and as few absences as possible. Although using thresholds based on specificity with presence-only data is problematic because it is impossible to determine if background points are true absences [50], for our purposes the true skill statistic offered an acceptable solution to the trade-off between maximising sensitivity and minimising predicted area (a condition necessary to focus fossil hunting) [51].

## Results

### Model validation and predictive performances

The three climate-envelope models we developed can accurately predict fossil occurrence, as shown by high median values of the true skill statistic and area under the receiver operator characteristic curve obtained by cross-validation (all values > 0.65 and 0.82, respectively; Table 1). The predictive performance of different models varied among genera. MaxEnt had the best performance for genera with small sample sizes, like *Zygomaturus* and *Thylacoleo*, whereas the generalised linear model had the worst (Table 1). For other genera, like *Genyornis*, the three models performed similarly.

**Table 1:** Climate-envelope models cross-validations.

Genus	Model	Spatio-temporal validation		Temporal validation	
		Median true skill statistic (1^st^–3^rd^ quartiles)	Median area under the receiver operator characteristic curve (1^st^–3^rd^ quartiles)	Median true skill statistic (1^st^–3^rd^ quartiles)	Median area under the receiver operator characteristic curve (1^st^–3^rd^ quartiles)
*Diprotodon*	Bioclim	0.78 (0.70–0.96)	0.90 (0.86–1.00)	0.98 (0.75–1.00)	1.00 (0.88–1.00)
	MaxEnt	0.78 (0.71–0.89)	0.93 (0.89–0.97)	0.97 (0.85–1.00)	0.98 (0.90–1.00)
	Generalised linear model	0.68 (0.63–0.74)	0.86 (0.81–0.90)	0.80 (0.63–1.00)	0.85 (0.80–1.00)
*Zygomaturus*	Bioclim	0.66 (0.50–0.96)	0.83 (0.75–0.99)	0.95 (0.00–1.00)	0.98 (0.50–1.00)
	MaxEnt	0.93 (0.86–0.96)	0.96 (0.93–0.99)	1.00 (0.95–1.00)	1.00 (0.95–1.00)
	Generalised linear model	0.79 (0.64–0.86)	0.82 (0.77–0.88)	0.90 (0.85–1.00)	0.90 (0.88–1.00)
*Protemnodon*	Bioclim	0.83 (0.69–0.86)	0.95 (0.89–1.00)	1.00 (0.80–1.00)	1.00 (0.90–1.00)
	MaxEnt	0.90 (0.84–0.95)	0.97 (0.94–0.98)	1.00 (0.95–1.00)	1.00 (0.95–1.00)
	Generalised linear model	0.73 (0.66–0.82)	0.87 (0.83–0.90)	0.90 (0.80–0.95)	0.90 (0.80–0.95)
*Thylacoleo*	Bioclim	0.80 (0.67–0.87)	0.89 (0.86–0.98)	1.00 (0.78–1.00)	1.00 (0.89–1.00)
	MaxEnt	0.94 (0.86–0.98)	0.98 (0.96–0.99)	1.00 (1.00–1.00)	1.00 (1.00–1.00)
	Generalised linear model	0.69 (0.62–0.77)	0.78 (0.74–0.82)	0.80 (0.70–0.90)	0.80 (0.70–0.90)
*Genyornis*	Bioclim	0.87 (0.84–0.90)	0.98 (0.97–0.99)	0.96 (0.88–1.00)	1.00 (0.95–1.00)
	MaxEnt	0.89 (0.87–0.92)	0.99 (0.98–0.99)	1.00 (0.90–1.00)	1.00 (0.98–1.00)
	Generalised linear model	0.85 (0.83–0.88)	0.94 (0.94–0.95)	0.91 (0.87–0.97)	0.95 (0.92–0.97)

Median (1st–3rd quartiles) of true skill statistic and area under the receiving operator characteristic curve for three climate-envelope models resulting from 100 rounds of 5-fold cross-validation.

Validation using independent data (i.e., unreliably dated fossils) showed that the projections of palaeo-climate suitability averaged through time predicted fossil occurrence better than random (median Kolmogorov-Smirnov statistics 0.43–0.75, median true skill statistics 0.43–0.75, Table 2). The model of fossil preservation had poor predictive capacity (median true skill statistic = 0.30; median area under the receiver operating curve = 0.63), but still performed better than random (mean Kolmogorov-Smirnov statistic = 0.34; S4 Table). The discovery model had only a slightly higher predictive capacity than the preservation model (median true skill statistic = 0.35; median area under the receiver operating curve = 0.67) and predictions were better than random (mean Kolmogorov-Smirnov statistic = 0.53; S4 Table).

**Table 2:** Data used to train climate-envelope models and validation steps for *Diprotodon*, *Zygomaturus*, *Protemnodon*, *Thylacoleo*, and *Genyornis*.

	Training			Validation		
Genus	Presences	Pseudo-absences	Age range (ka ago)	Presences	Median Kolmogorov-Smirnov statistic^a^ (1^st^–3^rd^ quartiles)	Median true skill statistic^b^ (1^st^–3^rd^ quartiles)
*Diprotodon*	23	230	44–112	14	0.43 (0.36–0.57)	0.43 (0.41–0.46)
*Zygomaturus*	14	140	44–120	9	0.67 (0.56–0.67)	0.45 (0.42–0.47)
*Protemnodon*	31	310	40–120	15	0.60 (0.53–0.67)	0.55 (0.53–0.58)
*Thylacoleo*	26	260	44–120	8	0.75 (0.75–0.88)	0.76 (0.74–0.79)
*Genyornis*	148	1312	36–120	8	0.63 (0.50–0.63)	0.55 (0.53–0.58)

In the training step, presence data describe fossils with reliable ages (black circles in Fig 3) whereas in the validation step, presences were fossils with unreliable ages (black crosses in Fig 3)

^a^ We did a Kolgomorov-Smirnov (KS) test compare the predicted suitabilities at fossil sites with the suitabilities predicted at 1000 sets of random points.

^b^ We calculated the True Skill Statistic (TSS) using unreliable fossils as independent presence points and 1000 sets of randomly selected pseudo-absences (10 times more than presences).

**Fig 2:**
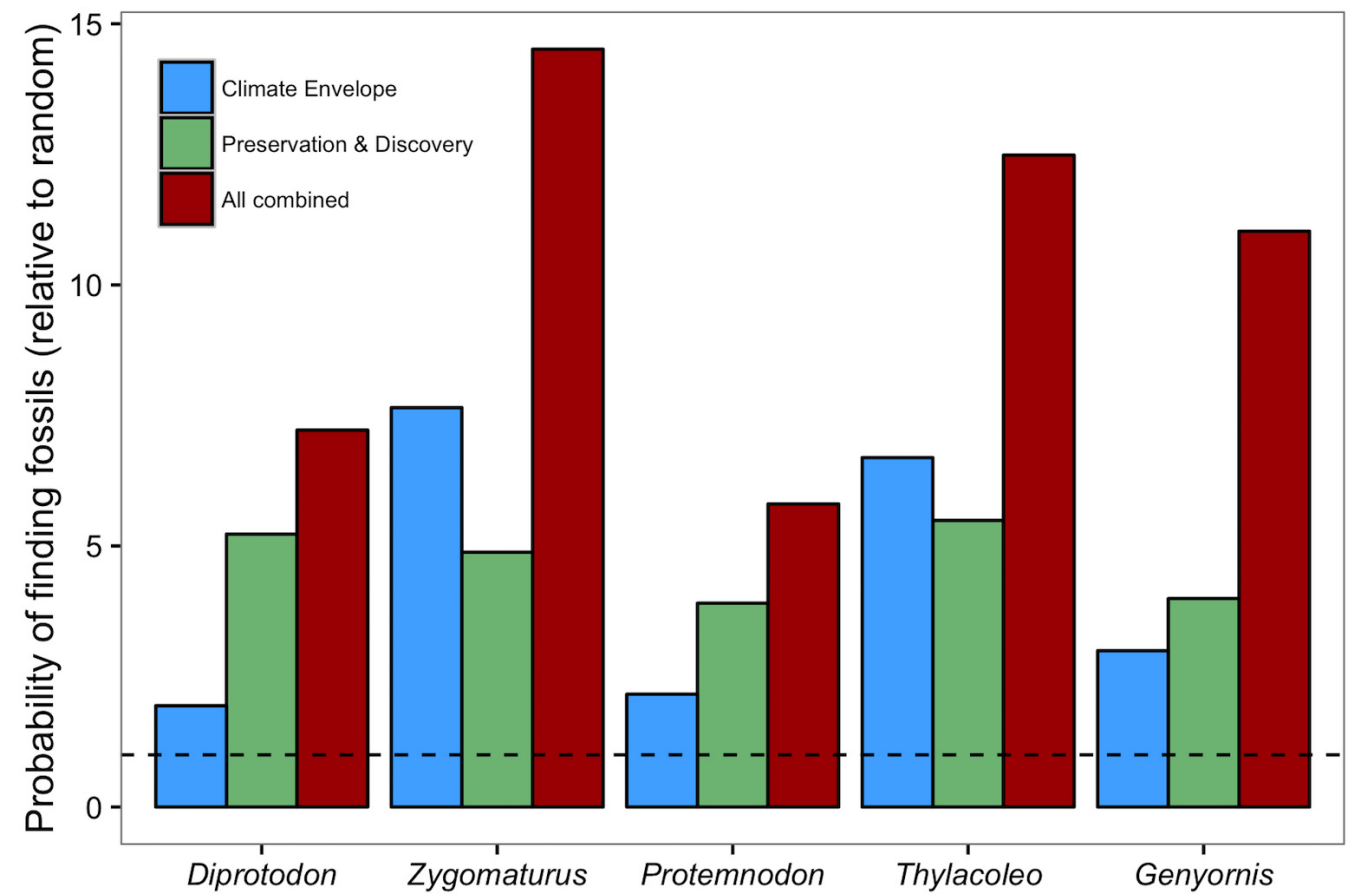
Effect of combining models on probability of finding fossils. Probability of finding a grid cell with independent fossil sites of five genera in areas predicted by the climate-envelope models (blue bars), in the area predicted by fossil preservation and discovery models (green bars), and in the area predicted by all models (i.e., climate-envelope, preservation, and discovery). Each probability is divided by the probability of finding the grid cells in all of Australia to emphasise usefulness of model combinations compared to finding fossils by chance. For example, a value of one (horizontal dashed line) would mean that the probability of finding a fossil using the model is the same as the probability of finding it by chance.

Averaging the output of the three models led to higher Kolmogorov-Smirnov values compared to estimates from separate models (S4 Table). The only exception was *Diprotodon*, for which the discovery model had a slightly higher median Kolmogorov-Smirnov statistic than the combined models (0.57 and 0.50, respectively). The probability of finding fossils of *Diprotodon*, *Protemnodon*, and *Genyornis* was higher in the overlapping areas suitable for fossil preservation and discovery, while it was higher in areas of suitable palaeo-climate for *Zygomaturus* and *Thylacoleo* (Fig 2). However, the highest probabilities for all genera were always where the three areas overlapped (S6 Fig).

### Model application

**Fig 3:**
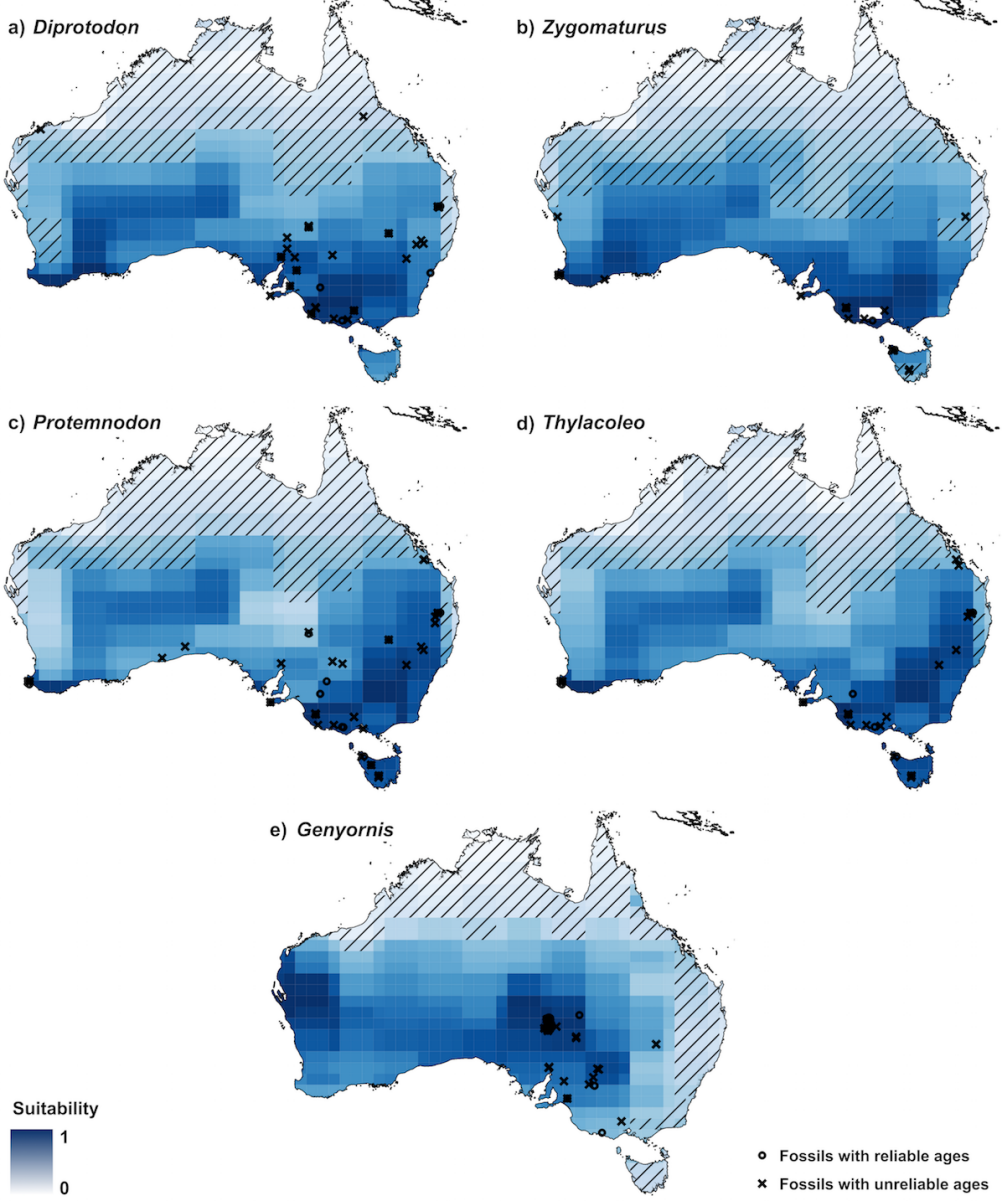
Multi-temporal climate suitability for *Diprotodon*, *Zygomaturus*, *Protemnodon*, *Thylacoleo*, and *Genyornis*. Maps display the climate suitability rankings (rescaled between 0 and 1) for each genus averaged across all time-slices during which the genus was still alive. Darker colours correspond to higher climate suitability. We used fossils with reliable ages (black circles) for model training and those without (black crosses) for validation. Diagonal lines indicate areas of extrapolation in model predictions (i.e., where there are values outside of the climate envelope used to train the model).

The projected climate suitabilities showed a similar pattern for all genera except *Genyornis* (Fig 3). The areas of highest suitability for *Diprotodon*, *Zygomaturus*, *Protemnodon*, and *Thylacoleo* were concentrated in south-eastern and south-western Australia (Fig 3A–3D). *Genyornis* had the most reliably dated fossils (148), but 93% were concentrated in the Lake Eyre region of central Australia. The projected area of highest suitability for *Genyornis* was the Lake Eyre basin and the climatically similar area of Western Australia near Shark Bay (Fig 3E), but it is unlikely that we captured the entire climate envelope of the genus.

**Fig 4:**
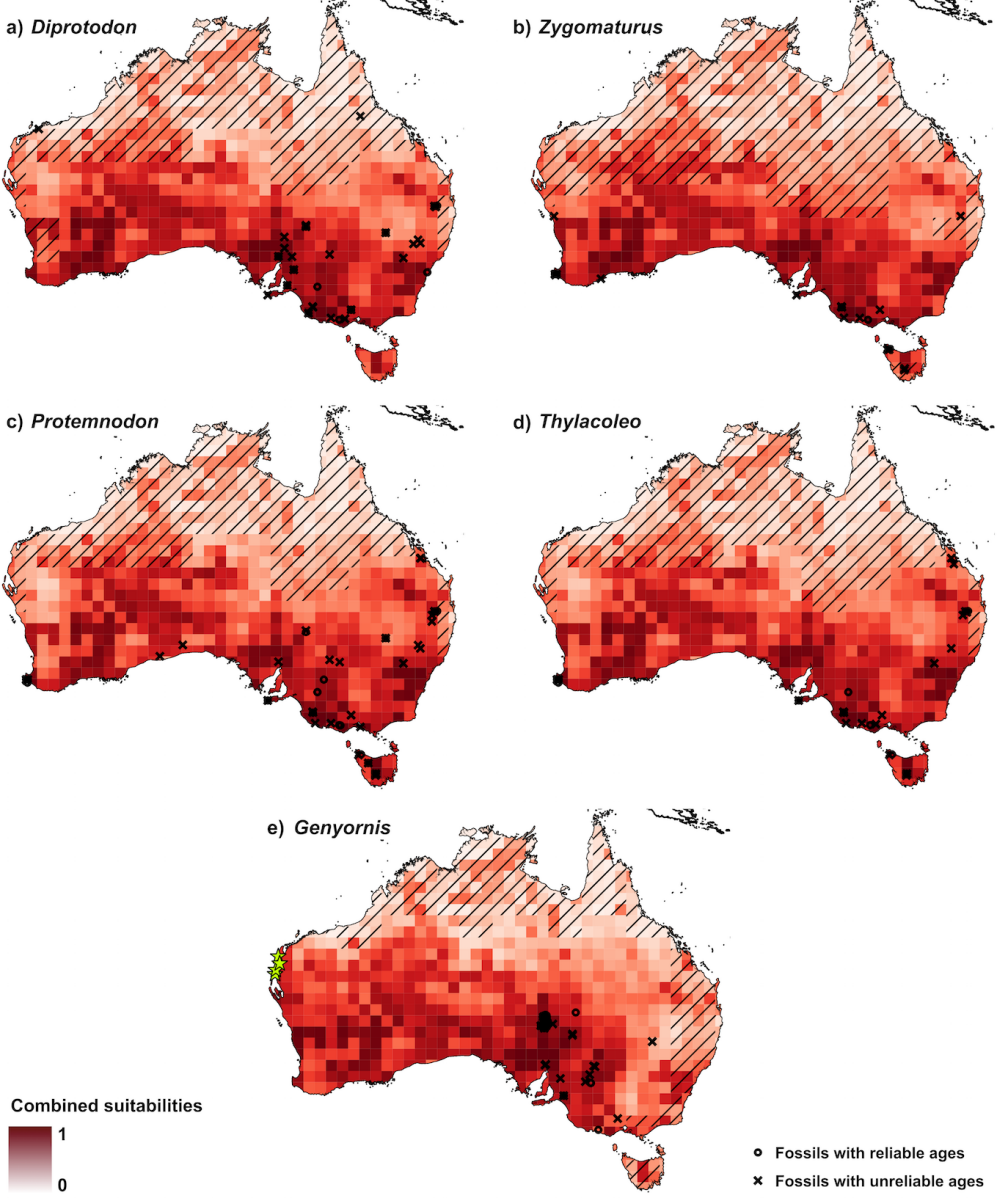
Combined-model prediction of where to search for fossils of *Diprotodon*, *Zygomaturus*, *Protemnodon*, *Thylacoleo*, and *Genyornis*. The places most likely to yield fossils of a given genus are the grid cells with the highest suitability. Maps display the climate suitability, suitability for fossil-preservation, and suitability for fossil-discovery rankings (rescaled between 0 and 1 and averaged) for each genus. Darker colours correspond to places more likely to yield new fossils. We used fossils with reliable ages (black circles) for climate-envelope model training and those without (black crosses) for validation of all models. Diagonal lines indicate areas of extrapolation in climate-envelope model predictions (i.e., where there are values outside of the climate envelope used to train the model). The yellow starts in map ‘e’ show the location of recent findings of new *Genyornis* eggshell remains [52], which provide an additional independent validation of our approach.

The areas with greatest potential for yielding new fossils of the four marsupial genera are concentrated in the southern half of mainland Australia and in central Tasmania (Fig 4). *Genyornis* was the only genus for which there are sites of good potential around Shark Bay, in Western Australia and around Lake Eyre, but not in the mountainous region of northern New South Wales (Fig 4E).

## Discussion

Combining climate-envelope, fossil preservation, and fossil discovery models is likely to improve the identification of new fossil-rich areas at continental scales. The highest probabilities of finding fossils are invariably at the intersection of the most suitable areas projected by the three models (Fig 2). This pattern is particularly strong for genera with spatially restricted data, such as *Genyornis* and *Zygomaturus*, for which areas of suitable climate differed from areas suitable for fossil discovery and preservation (S6 Fig).

In contrast to recent modelling approaches applied to fossil hunting [7,9–11,49], our method predicts potential fossil locations across an entire continent, which is useful to identify potential fossils areas far from already known sites. Despite having low spatial resolution (1 × 1°), our method narrowed the potential areas of interest more effectively than picking locations by chance (Fig 2). As such, combined with the expertise of palaeontologists, our method is a good initial ‘exploration filter’ for identifying potential fossil areas, after which remote-sensing approaches (e.g., [10,11]) and fine-scale expert knowledge could complement the search.

Our approach revealed several areas with a higher-than-random potential of yielding new fossils. In South Australia, south of Lake Eyre and west of Lake Torrens, there is an area of high potential to yield new fossils for all the genera we examined, especially for *Diprotodon* (Figs 3 and 4). For *Genyornis*, there is a large area in western Australia around Shark Bay where palaeo-climate suitability is high (Fig 3); there has recently been a discovery of new *Genyornis* eggshell remains in that region [52], thus providing an independent confirmation of our approach. For *Diprotodon*, *Zygomaturus*, *Protemnodon* and *Thylacoleo*, there are also several grid cells with high fossil-yielding potential in south-western Australia (Fig 4A–4D). All these areas, and especially the last two, are far from all known fossil sites, and hence it is unlikely that they would have been identified as potential sites based on traditional fossil-hunting approaches.

A taxon’s palaeo-distribution is relevant for fossil hunting and it might be the best single indicator of where to look for its fossils at continental scales (Fig 2). The probability of finding grid cells with fossils in areas of suitable palaeo-climate was more than twice the probability of finding them over the entire grid of Australia, and was nearly the same as in the areas with the highest potential for fossil preservation and discovery (i.e., where previous modelling approaches to fossil hunting have focused) [9–11,49].

Areas with the highest climatic suitabilities for all the genera we examined were mainly in the southern half of Australia. This might reflect true climatic suitability for the genera we examined here, but we cannot entirely discount taphonomic and sampling biases in the fossil records used to train the models. There are fossil records of *Diprotodon*, *Protemnodon*, and *Thylacoleo* in the Australian tropics but we could not include them in the climate-suitability models because their age estimates are unreliable [13]. Another limitation of our method is that our estimation of a taxon’s climate envelope is based on the known fossils with reliable ages, and thus it will do poorly at predicting fossil sites in different climates were the taxon could have lived. To avoid this we would have to use a mechanistic model based on the taxon’s inferred climatic tolerances [53]. The climate suitability probably represents an unknown combination of each taxon’s true climate envelope *and* of the likelihood of fossil preservation and discovery [12]. Although this would be undesirable if the main objective was to quantify the true palaeo-distributions of each taxon, such biases could in fact be advantageous for improving the probability of fossil discovery.

The predictive capability of the climate-envelope models is remarkable (median true skill statistic = 0.43–0.76, Table 2) considering that we only used mean annual temperature and precipitation as predictors. Including non-climatic environmental information such as topography could improve model performance [54]. Considering biotic interactions could further improve model accuracy [55]. Interactions with humans strongly modified the realised distributions of many megafauna species of Australia’s Late Pleistocene [21], an association that is not explicitly captured by our models. For example, *Genyornis newtoni* occurred sympatrically over much of its climatic range with *Dromaius novaehollandiae* (emu) until around 36 ka ago, when *G*. *newtoni* went extinct while *D*. *novaehollandiae* persisted (S7A Fig) [56]. Since the climate envelopes of both species overlapped considerably (S7B Fig), our results suggest that something other than climate (i.e., annual temperature and precipitation), such as human hunting, lead to a rapid contraction of *Genyornis*’ distribution [52,56].

Our three-step method could easily be modified to assist fossil-site identification on any continent. We show that the area of suitable palaeo-climate for taxa (or fossil) occurrence can be successfully modelled with as few as 14 records from different points in space and time (e.g., *Zygomaturus*). For small sample sizes, MaxEnt performs particularly well, in agreement with previous findings [29,31]. Genetic algorithms have been used to model palaeo-distributions with as few as five fossil records [57], so they could be incorporated into the method to deal with small sample sizes. We gave the same weight to the climate-envelope, preservation, and discovery models when calculating the final likelihood of finding fossils, but weighting models by their predictive performance could potentially yield better results in some circumstances. Global circulation model-based palaeo-climate reconstructions constrain the temporal window to which the method can be applied. At this stage, the method is only useful for identifying potential fossil areas of Late Pleistocene and early Holocene fauna. Using other proxies of environmental conditions could potentially adapt the method for use with older fossils [18,57]. As suitable climate proxies and reconstructions pierce ever-backward in time [58,59], the capacity to model palaeo-distributions of both extinct and extant species will become more powerful and ecologically realistic [18].

## Supporting Information

S1 Appendix Description of methods used to model climate envelopes. (PDF)

S1 Fig **Spatial (a) and temporal (b) distribution of fossils used to train and validate climate-envelope models for *Diprotodon*, *Zygomaturus*, *Protemnodon*, *Thylacoleo*, and *Genyornis*.** For model training, we used only fossils with reliable ages (black circles and red grid cells in a)[14]. For validation, we used grid cells that had only unreliably dated fossils (black crosses and blue grid cells in a). Each cross in b represents the estimated age of a fossil, and the line is a confidence interval of one standard deviation. Crosses are randomly spread away from the line to show the density of fossil records at different times.
(PDF)

S2 Fig Map of fossil sites (a) and density of fossils per grid cell (b).
(PDF)

S3 Fig Maps of variables used in the fossil-preservation model. We considered sedimentary rocks and regoliths as suitable for fossil preservation (a), and calculated their area in each grid cell (b). The large amounts of sediments transported by changes in water level in lakes (c) facilitate the burial of dead organisms and their subsequent fossilisation. Hence, we calculated the area of lakes in each grid cell (d). Caves serve as pitfall traps (e), so we used the presence/absence of caves in each grid cell (f) as a binary predictor of its suitability for fossil preservation (grey = suitable and white = unsuitable).
(PDF)

S4 Fig Maps of variables used in the fossil-discovery model. We used maps of slope across Australia (a), bare soil (c) and rain intensity (annual rainfall divided by annual days of rain, e) and calculated their values in each grid cell (b,d,f). Areas of steep slope are represented by white in ‘a’ and by dark reds in ‘b’. Areas of bare soil are shown in red in ‘c’. In ‘d’, grid cells with darker colours have larger areas of bare soil. In ‘e’, high and low values of annual rainfall are represented with yellow and green, respectively, while white represents areas with more days of rain per year. In ‘f’, darker blues show grid cells with higher values of rain intensity.
(PDF)

S5 Fig **Maps of the suitability for fossil preservation (a) and discovery (b).** Fossil-preservation suitability is a function of the presence of caves and the cover of lakes and suitable rocks per grid cell. Fossil-discovery suitability, corrected for sampling bias, is a function of erosion proxies: mean rain intensity, mean slope, and cover of bare soil per grid cell. Suitability values were ranked and rescaled between 0 and 1. Darker colours represent higher suitabilities. Black crosses represent fossil sites.
(PDF)

S6 Fig Maps of the overlap of suitability areas predicted by climate-envelope, preservation and discovery models for *Diprotodon*, *Zygomaturus*, *Protemnodon*, *Thylacoleo*, and *Genyornis*. These areas are the result of converting the continuous output into binary (presence/absence), using a threshold that maximised the true skill statistic. Hence, if a grid cell is outside the ‘presence’ area, it is still possible to find fossils there. Even if average conditions across the grid cell area are not optimal for finding fossils, there still might be a place where the right conditions exist. Rather, the binary output shows the grid cells where palaeo-climate history and conditions associated with fossil preservation and discovery are optimal. The chances of finding fossils in this area are higher than in any other randomly selected grid cell, and thus it is there where future fossil hunting could focus.
(PDF)

S7 Fig Climate envelopes of *Genyornis newtoni* and *Dromaius novaehollandiae*. (a) Comparison of climate-envelope dynamics at 56, 46, 34 and 32 ka ago. Circles show fossil locations for each species and darker reds represent higher climatic suitabilities. (b) Overlap of climate-envelopes of both species.
(PDF)

S1 Table Generalised linear models ranked by information criterion. (PDF)

S2 Table Summary of fossil preservation model. Estimated coefficients of logistic regression of fossil occurrence as a function of the presence of caves, area of lakes, and area of rocks suitable for fossil preservation.
(PDF)

S3 Table Summary of fossil discovery model. Estimated coefficients of a logistic regression of fossil occurrence as a function of the area of bare soil, mean rain intensity, and mean slope.
(PDF)

S4 Table Validation results for the predictions of the single climate-envelope, preservation, and discovery models, as well as their combined predictions. We compared the suitability values predicted for grid cells containing fossils with unreliable ages of a given genus (not used to train the models) with the suitability values predicted for 1000 sets of randomly selected grid cells from across Australia using a Kolmogorov-Smirnov test.
(PDF)
