# Supplementary material for: Where to Dig for Fossils: Combining Climate-Envelope, Taphonomy and Discovery Models
Source: PLoS One. 2016 Mar 30;11(3):e0151090. doi: 10.1371/journal.pone.0151090 (PMC4814095; doi:10.1371/journal.pone.0151090)
Supplement: S1 Appendix — (PDF) [file pone.0151090.s001.pdf]

S1 Appendix. Description of climate-envelope models: presence-only (BIOCLIM), presence-background (MaxEnt), and presence-(pseudo)absence (generalised linear model).

Climate-envelope models attempt to capture the climatic conditions that constrain the potential niche of a species, and use them to predict the probability of occurrence of species in an area. There are many different types of climate-envelope models [1], distinguished among other things by the type of data they use and the type of predictions that can make [2]. The performance of climate-envelope models can also vary depending on the characteristics of the data [3–5]. Thus, we applied three different types of models, each with its own strengths and weaknesses, and then averaged their predictions weighted by their respective predictive performances (evaluated with the true skill statistic).

BIOCLIM was one of the first algorithms used to model species distributions [6], and is one of a small family of climate-envelope models that rely only on presence data (fossils are necessarily presence-only data). Other methods require the use of 'background' or absence data (known or suspected points in space where the species is/was absent), but BIOCLIM simply describes the climate envelope of a species as the multidimensional niche between the extreme conditions in which the species has been observed. It can rank the suitability of an area as a function of how far the climatic conditions of a site are from the median climatic conditions in which the species has been observed. Because it is simple and requires few assumptions compared to other methods, it has been recommended for modelling palaeo-distributions based on fossil records [7].

MaxEnt [8] has become one of the most popular methods to model species distributions with presence-only data due to its general good performance [9]. The method is based in the principle of maximum entropy, which states that from an infinity of possible climate envelopes with a common set of constraints, the one with the maximum entropy should be preferred (see refs 9 and 10 for a detailed statistical description of MaxEnt). For example, constraints could be that the climate envelope of a species should have the same mean or median as the climates in which the species was observed. However, there is an infinite number of different climate envelopes that produce the same median or mean. Thus, of all the possibilities, the one with the maximum entropy (i.e*.,* closest to a uniform distribution) should prevail. To determine which climate envelope has maximum entropy, the climates in which the species has been observed as well as the availability of climates throughout the study region must be known. As such, MaxEnt is called a *presence-background* method, in contrast to BIOCLIM.

The binomial generalised linear model represents a powerful technique to model not only the occurrence of species, but almost any phenomenon with a binary response [11]. In contrast to BIOCLIM and MaxEnt however, generalised linear models require presence *and* absence data. While the background data used by MaxEnt are not assumed to be places where the species is truly absent, the absences used in generalised linear models are. This can be troublesome even when absences have been recorded during field surveys [12], and even more so when true absence data are lacking. Nonetheless, it is common to select pseudo-absences from the background area for generalised linear models [13,14]. Pseudo-absences are points where the species has not been observed and are assumed to be absences. Selecting pseudo-absences in a way that minimizes the risk of having false absences (e.g., by selecting them from places with climates in which the species has not been observed) or that accounts for spatial biases in the sampling effort can improve the performance of climate-envelope models [14], but sometimes simply picking pseudo-absences randomly results in the best predictions [15].

References

1. Elith J, Graham CH, Anderson RP, Dudik M, Ferrier S, Guisan A, et al. Novel methods improve prediction of species' distributions from occurrence data. Ecography. 2006;29: 129–151. doi:10.1111/j.2006.0906-7590.04596.x

2. Guillera-Arroita G, Lahoz-Monfort J, Elith J, Gordon A, Kujala H, Lentini P, et al. Is my species distribution model fit for purpose? Matching data and models to applications. Glob Ecol Biogeogr. 2015; 276–292. doi:10.1111/geb.12268

3. Miller J. Virtual species distribution models: Using simulated data to evaluate aspects of model performance. Prog Phys Geogr. 2014;38: 117–128. doi:10.1177/0309133314521448

4. Stockwell DRB, Peterson AT. Effects of sample size on accuracy of species distribution models. Ecol Modell. 2002;148: 1–13. doi:10.1016/S0304-3800(01)00388-X

5. Elith J, Leathwick JR. Species Distribution Models: Ecological Explanation and Prediction Across Space and Time. Annu Rev Ecol Evol Syst. 2009;40: 677–697. doi:10.1146/annurev.ecolsys.110308.120159

6. Booth TH, Nix HA, Busby JR, Hutchinson MF. Bioclim: The first species distribution modelling package, its early applications and relevance to most current MaxEnt studies. Divers Distrib. 2014;20: 1–9. doi:10.1111/ddi.12144

7. Varela S, Lobo JM, Hortal J. Using species distribution models in paleobiogeography: A matter of data, predictors and concepts. Palaeogeogr Palaeoclimatol Palaeoecol. 2011;310: 451–463.

doi:10.1016/j.palaeo.2011.07.021

8. Phillips SJ, Anderson RP, Schapire RE. Maximum entropy modeling of species geographic distributions. Ecol Modell. 2006;190: 231–259. doi:10.1016/j.ecolmodel.2005.03.026

9. Merow C, Smith MJ, Silander JA. A practical guide to MaxEnt for modeling species' distributions: What it does, and why inputs and settings matter. Ecography. 2013;36: 1058–1069. doi:10.1111/j.1600-0587.2013.07872.x

10. Elith J, Phillips SJ, Hastie T, Dudík M, Chee YE, Yates CJ. A statistical explanation of MaxEnt for ecologists. Divers Distrib. 2011;17: 43–57. doi:10.1111/j.1472-4642.2010.00725.x

11. Austin MP. Spatial prediction of species distribution: an interface between ecological theory and statistical modelling. Ecol Modell. 2002;157: 101–118. doi:10.1016/S0304-3800(02)00205-3

12. Lobo JM, Jiménez-Valverde A, Hortal J. The uncertain nature of absences and their importance in species distribution modelling. Ecography. 2010;33: 103–114. doi:10.1111/j.1600-0587.2009.06039.x

13. Elith J, Leathwick J. Predicting species distributions from museum and herbarium records using multiresponse models fitted with multivariate adaptive regression splines. Divers Distrib. 2007;13: 265–275. doi:10.1111/j.1472-4642.2007.00340.x

14. Iturbide M, Bedia J, Herrera S, del Hierro O, Pinto M, Gutiérrez JM. A framework for species distribution modelling with improved pseudo-absence generation. Ecol Modell. 2015;312: 166–174. doi:10.1016/j.ecolmodel.2015.05.018

15. Sequeira A, Mellin C, Rowat D, Meekan MG, Bradshaw CJA. Ocean-scale prediction of whale shark distribution. Divers Distrib. 2012;18: 504–518. doi:10.1111/j.1472-4642.2011.00853.x
