# Supplementary figures and images for: Where to Dig for Fossils: Combining Climate-Envelope, Taphonomy and Discovery Models
Source: PLoS One. 2016 Mar 30;11(3):e0151090. doi: 10.1371/journal.pone.0151090 (PMC4814095; doi:10.1371/journal.pone.0151090)

**a**

*Diprotodon*

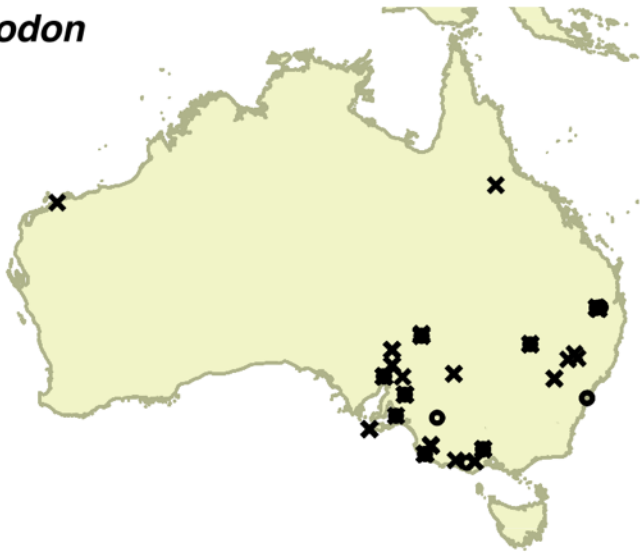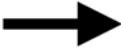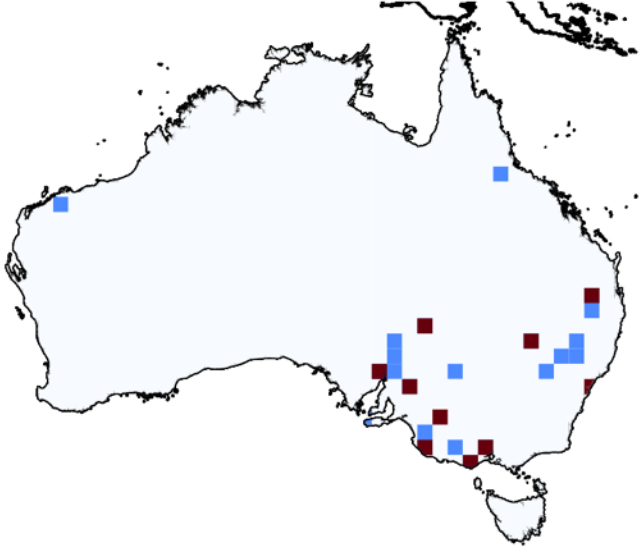

*Zygomaturus*

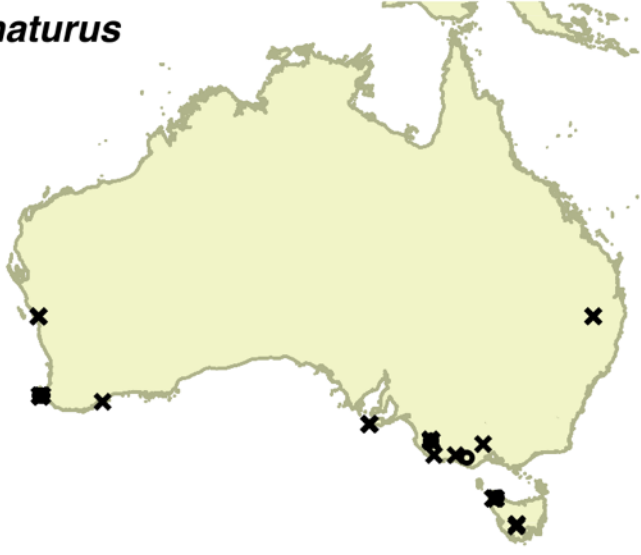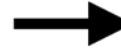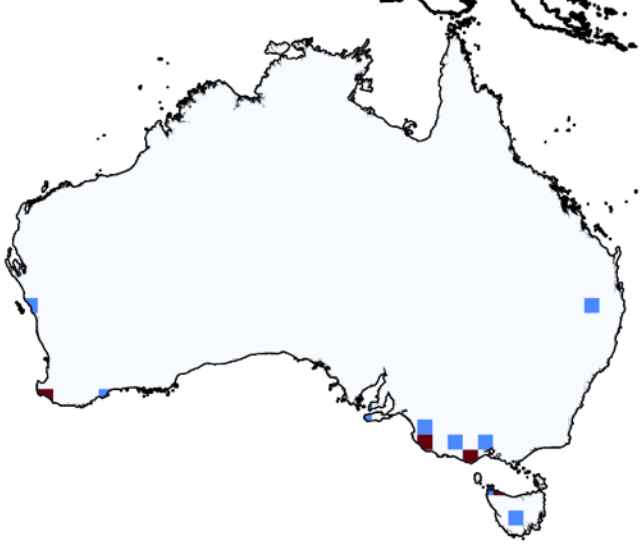

*Protemnodon*

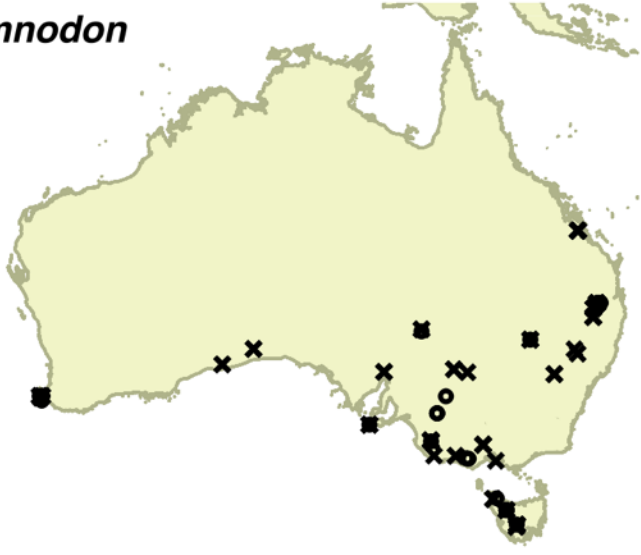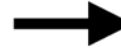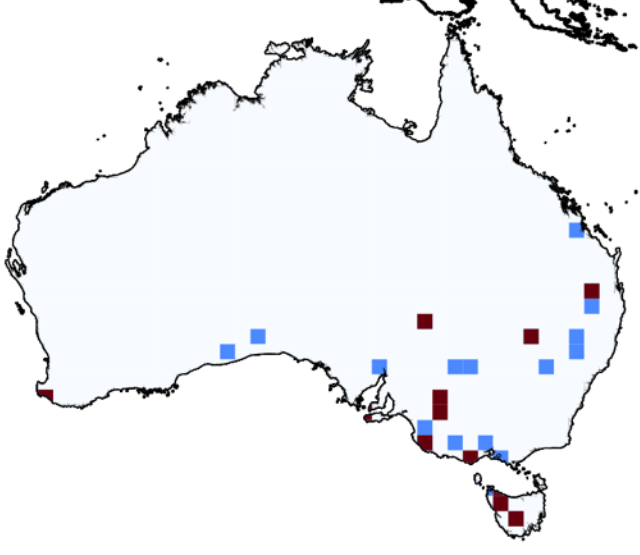

*Thylacoleo*

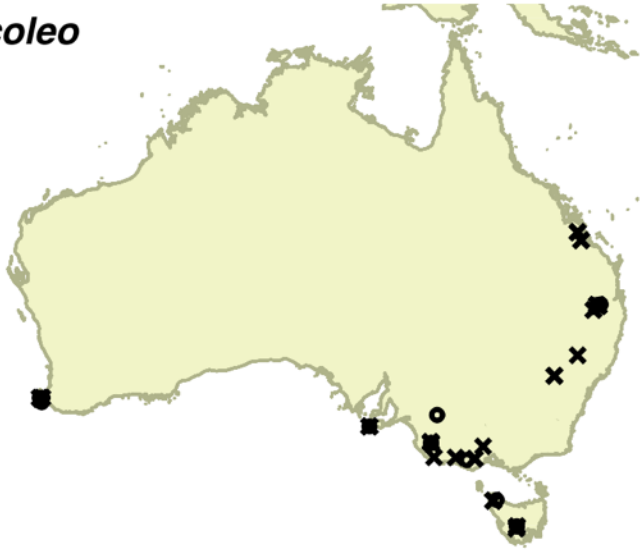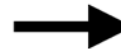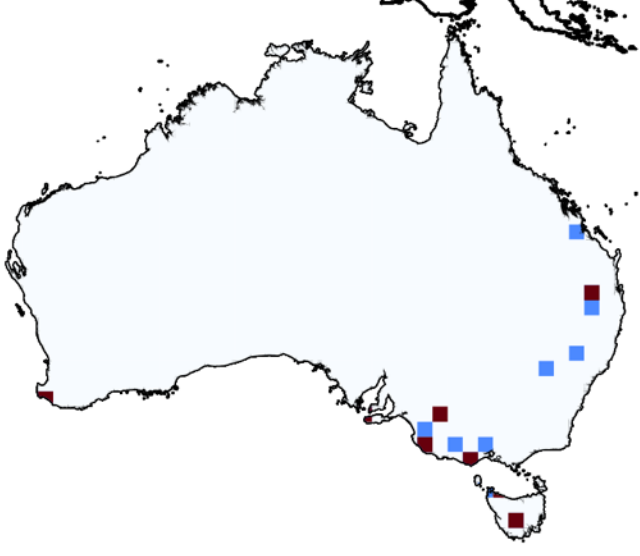

*Genyornis*

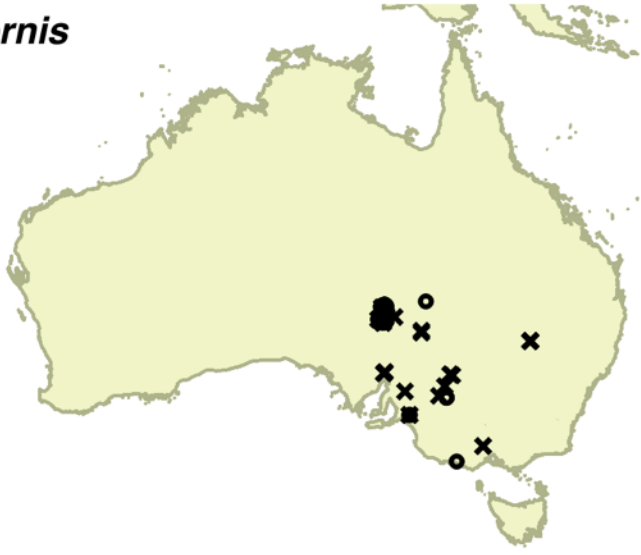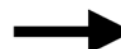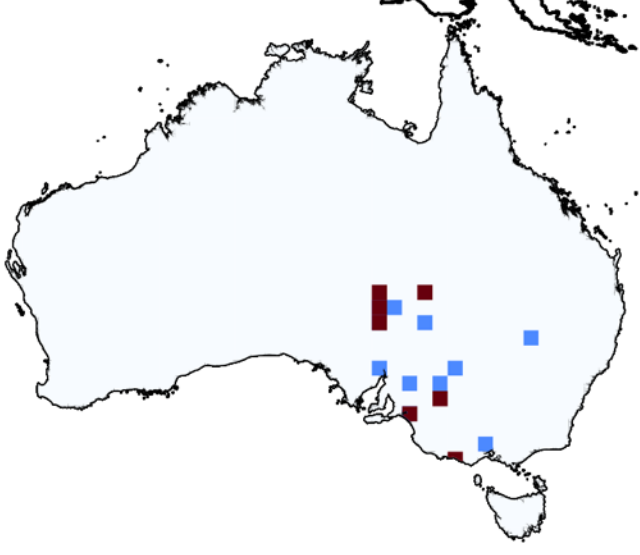

**b**

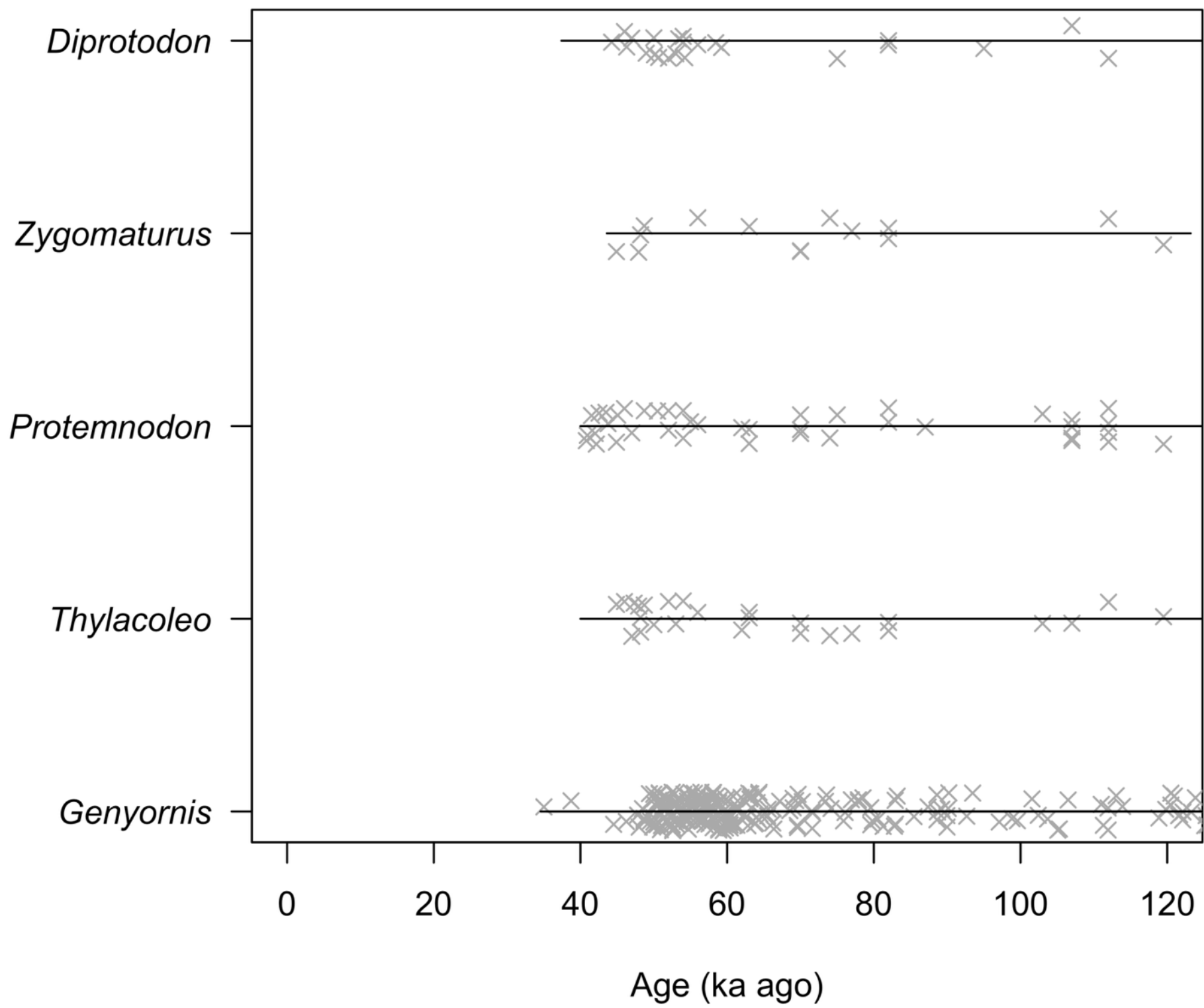

Supplement: S1 Fig — Spatial (a) and temporal (b) distribution of fossils used to train and validate climate-envelope models for Diprotodon, Zygomaturus, Protemnodon, Thylacoleo, and Genyornis. For model training, we used only fossils with reliable ages (black circles and red grid cells in a)[14]. For validation, we used grid cells that had only unreliably dated fossils (black crosses and blue grid cells in a). Each cross in b represents the estimated age of a fossil, and the line is a confidence interval of one standard deviation. Crosses are randomly spread away from the line to show the density of fossil records at different times. (PDF) [file pone.0151090.s002.pdf]

a

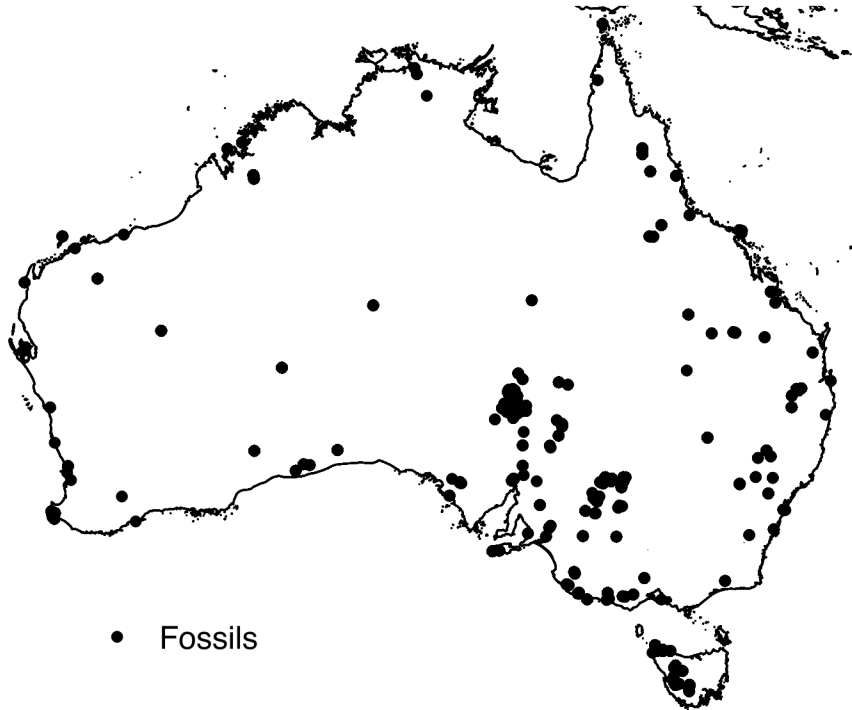

b

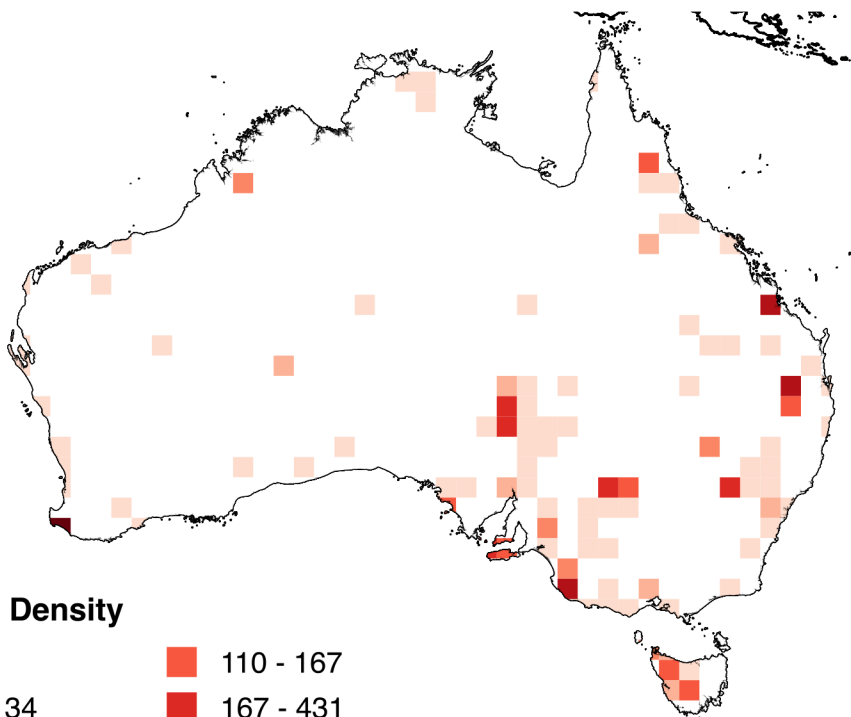

### Fossil Density

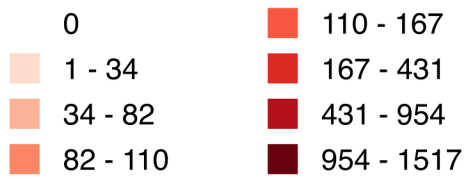

Supplement: S2 Fig — Map of fossil sites (a) and density of fossils per grid cell (b). (PDF) [file pone.0151090.s003.pdf]

## Lithology

a

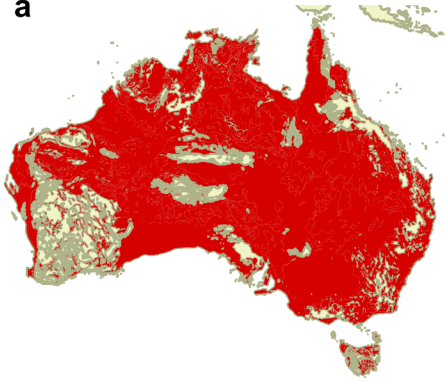

b

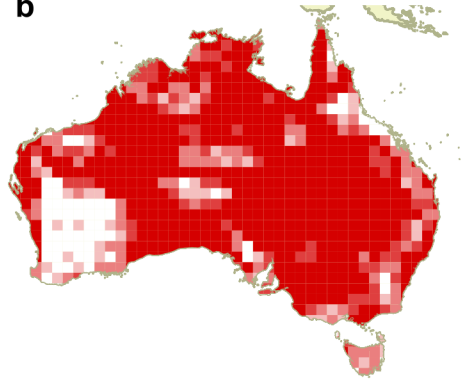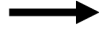

## Lakes

c

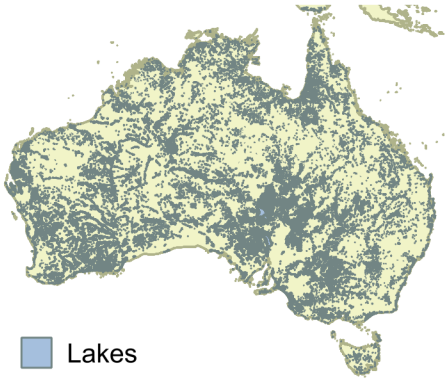

d

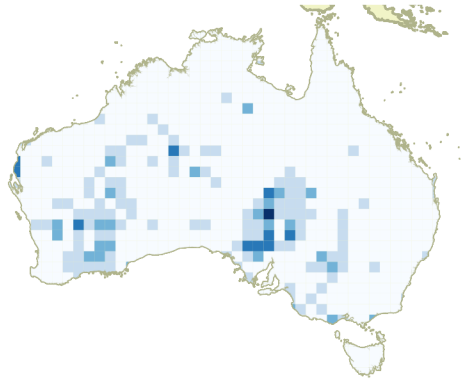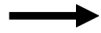

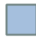 Lakes

## Caves

e

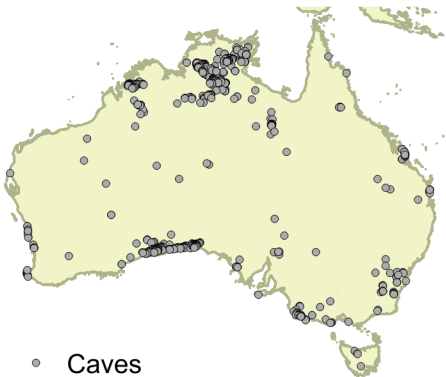

f

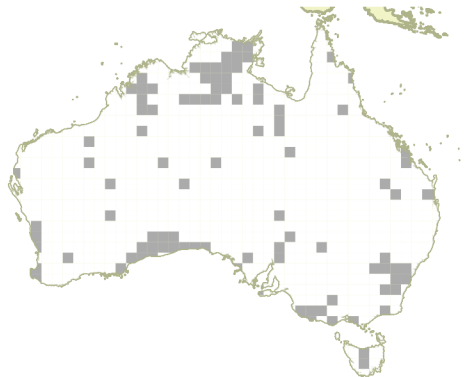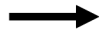

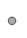 Caves

Supplement: S3 Fig — We considered sedimentary rocks and regoliths as suitable for fossil preservation (a), and calculated their area in each grid cell (b). The large amounts of sediments transported by changes in water level in lakes (c) facilitate the burial of dead organisms and their subsequent fossilisation. Hence, we calculated the area of lakes in each grid cell (d). Caves serve as pitfall traps (e), so we used the presence/absence of caves in each grid cell (f) as a binary predictor of its suitability for fossil preservation (grey = suitable and white = unsuitable). (PDF) [file pone.0151090.s004.pdf]

## Slope

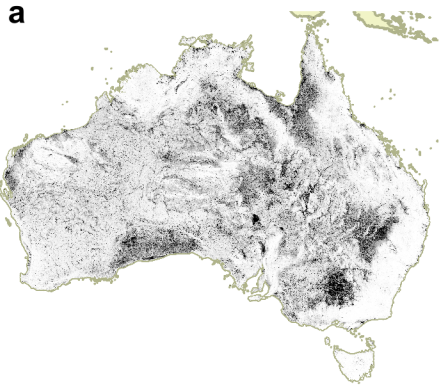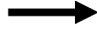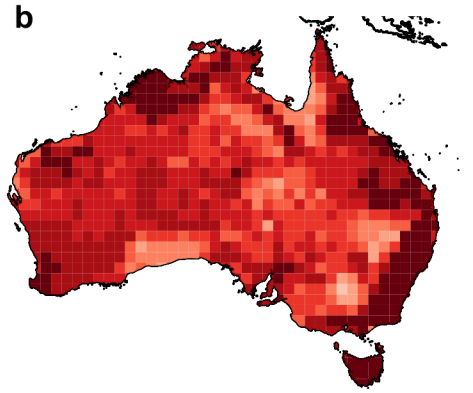

## Bare soil

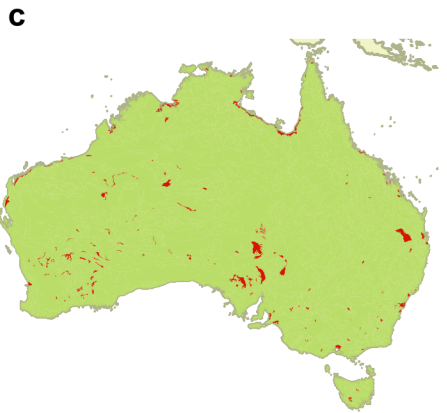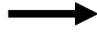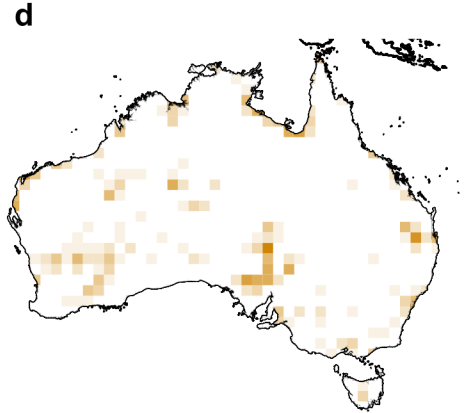

## Rain intensity

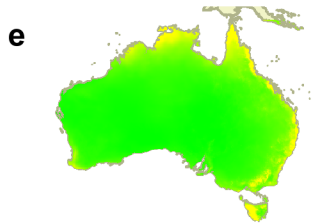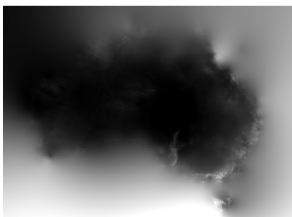

Annual rainfall

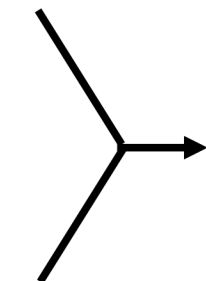

Annual days of rain

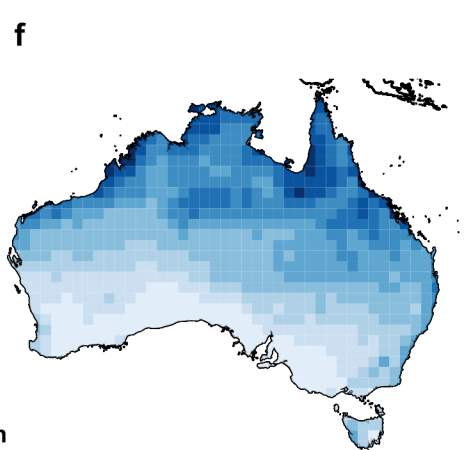

Supplement: S4 Fig — We used maps of slope across Australia (a), bare soil (c) and rain intensity (annual rainfall divided by annual days of rain, e) and calculated their values in each grid cell (b,d,f). Areas of steep slope are represented by white in ‘a’ and by dark reds in ‘b’. Areas of bare soil are shown in red in ‘c’. In ‘d’, grid cells with darker colours have larger areas of bare soil. In ‘e’, high and low values of annual rainfall are represented with yellow and green, respectively, while white represents areas with more days of rain per year. In ‘f’, darker blues show grid cells with higher values of rain intensity. (PDF) [file pone.0151090.s005.pdf]

a) Preservation

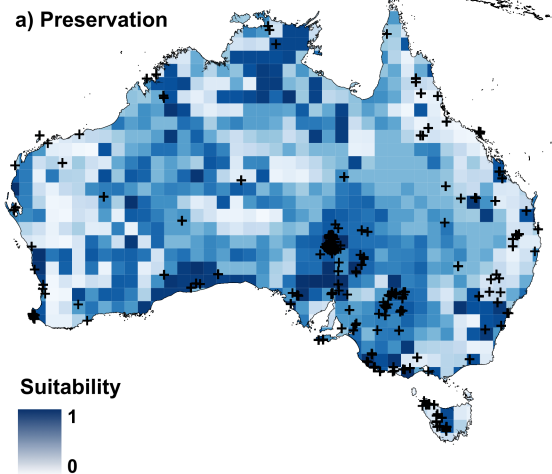

b) Discovery

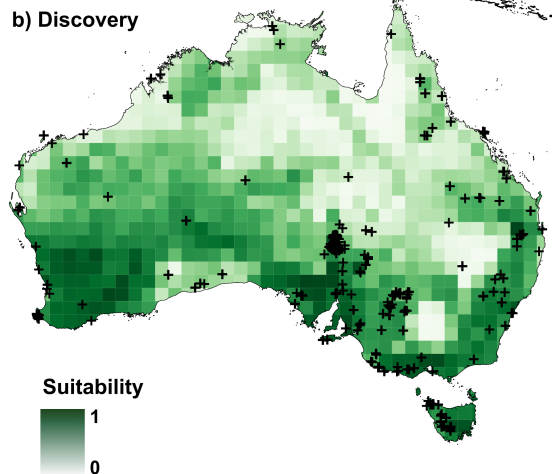

Supplement: S5 Fig — Maps of the suitability for fossil preservation (a) and discovery (b). Fossil-preservation suitability is a function of the presence of caves and the cover of lakes and suitable rocks per grid cell. Fossil-discovery suitability, corrected for sampling bias, is a function of erosion proxies: mean rain intensity, mean slope, and cover of bare soil per grid cell. Suitability values were ranked and rescaled between 0 and 1. Darker colours represent higher suitabilities. Black crosses represent fossil sites. (PDF) [file pone.0151090.s006.pdf]

*Diprotodon*

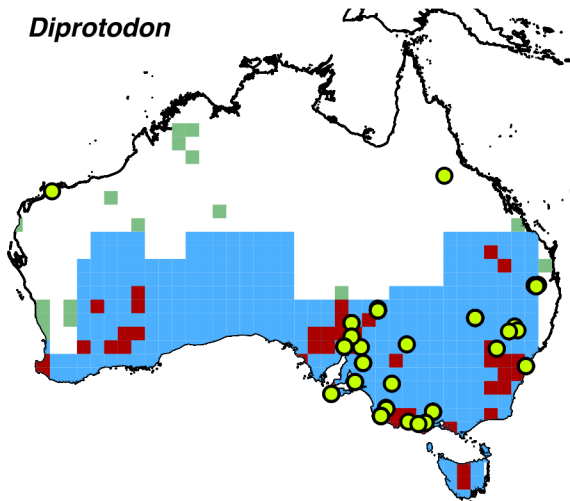

*Zygomaturus*

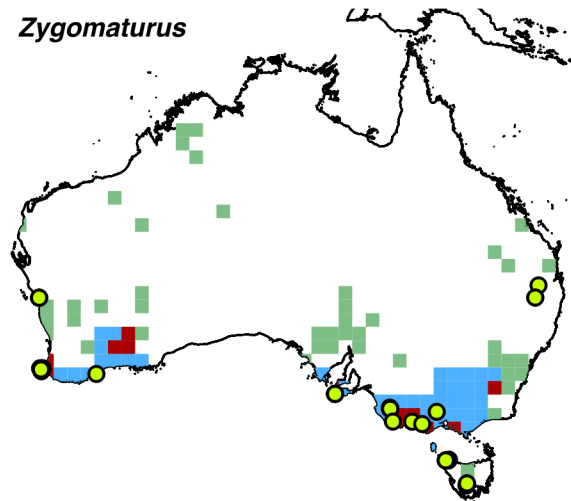

*Protemnodon*

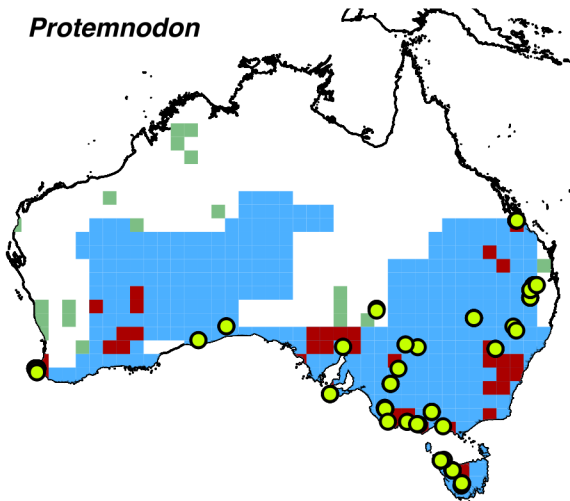

*Thylacoleo*

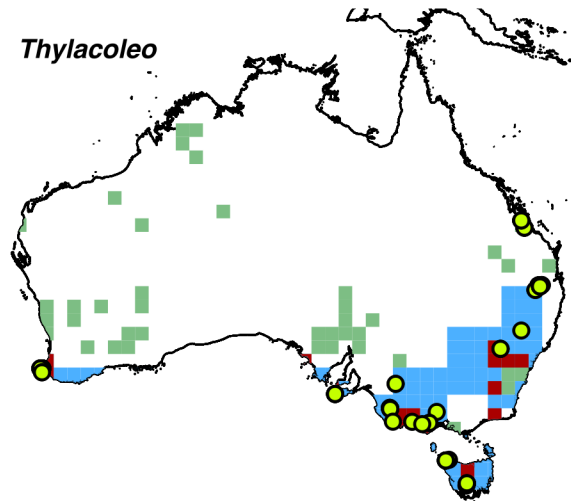

*Genyornis*

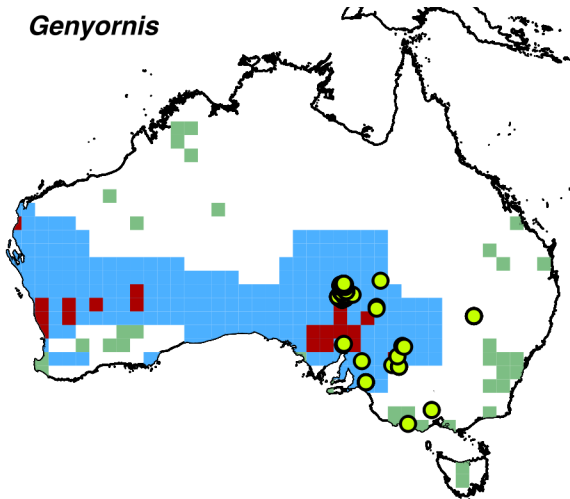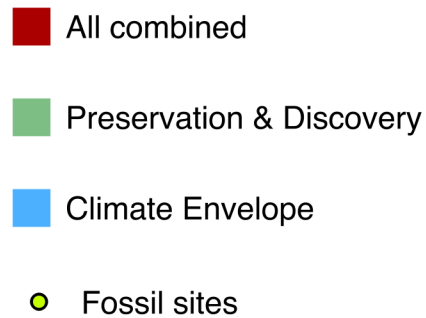

Supplement: S6 Fig — These areas are the result of converting the continuous output into binary (presence/absence), using a threshold that maximised the true skill statistic. Hence, if a grid cell is outside the ‘presence’ area, it is still possible to find fossils there. Even if average conditions across the grid cell area are not optimal for finding fossils, there still might be a place where the right conditions exist. Rather, the binary output shows the grid cells where palaeo-climate history and conditions associated with fossil preservation and discovery are optimal. The chances of finding fossils in this area are higher than in any other randomly selected grid cell, and thus it is there where future fossil hunting could focus. (PDF) [file pone.0151090.s007.pdf]

**a**

*Genyornis newtoni*

*Dromaius novaehollandiae*

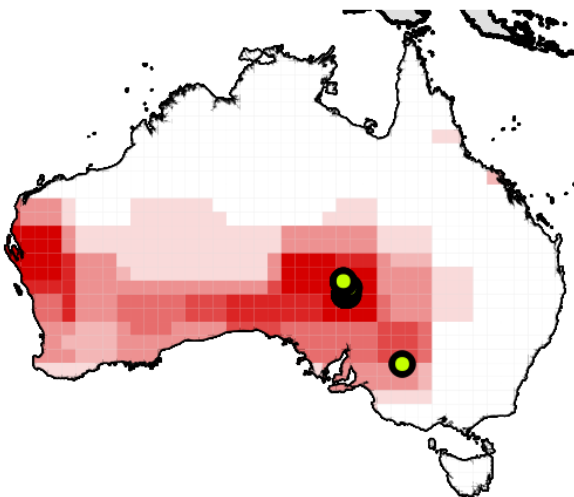

56 ka

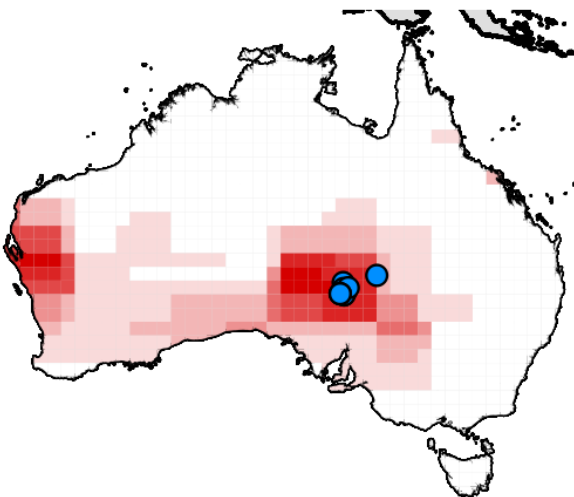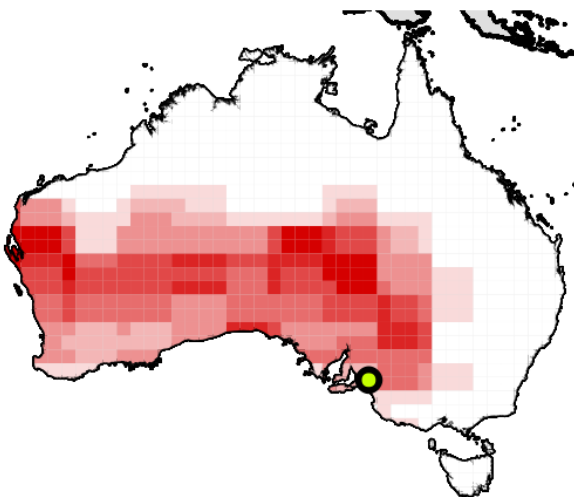

46 ka

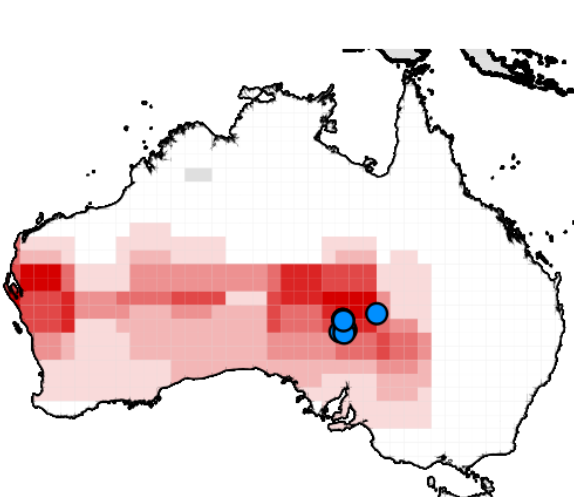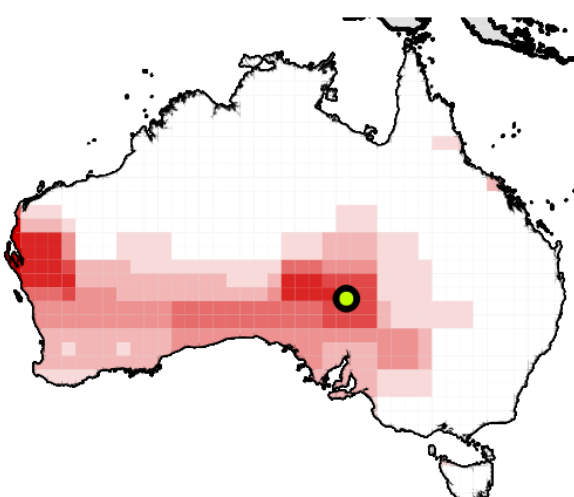

36 ka

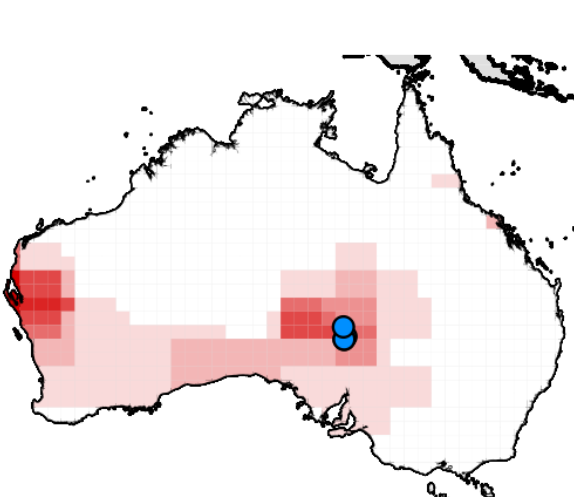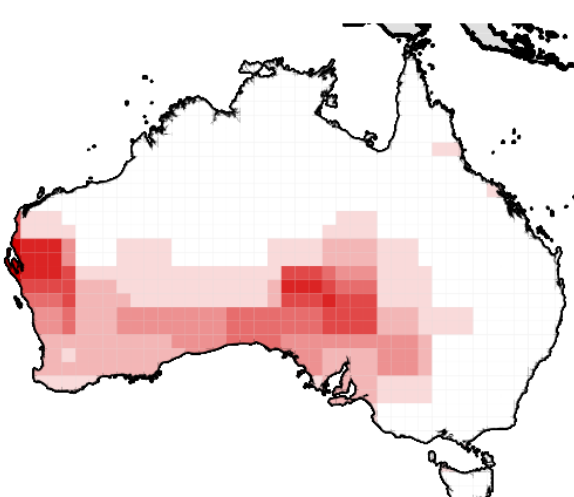

34 ka

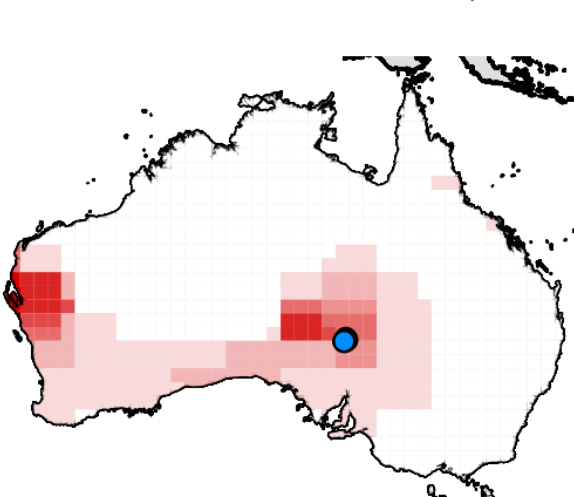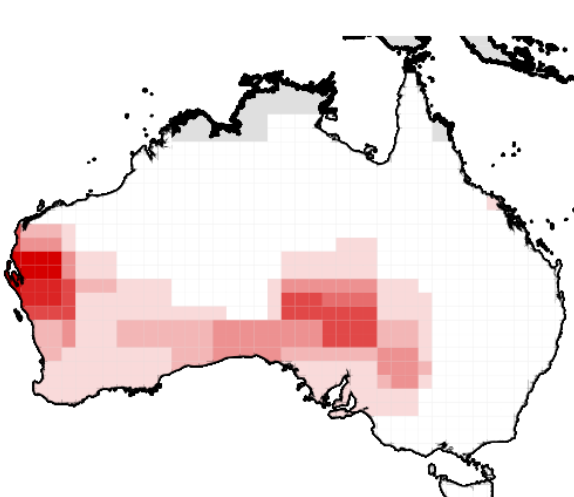

32 ka

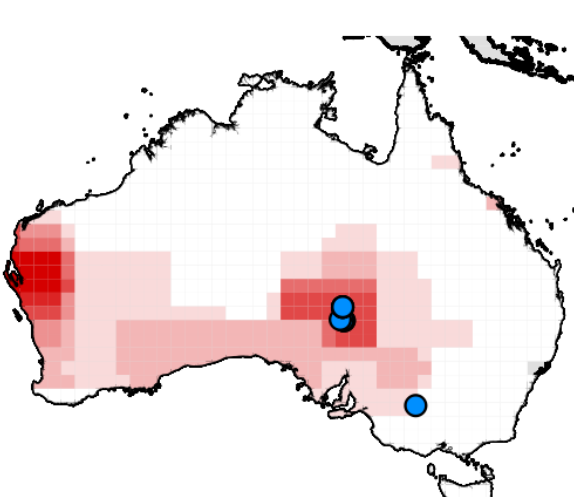

**b**

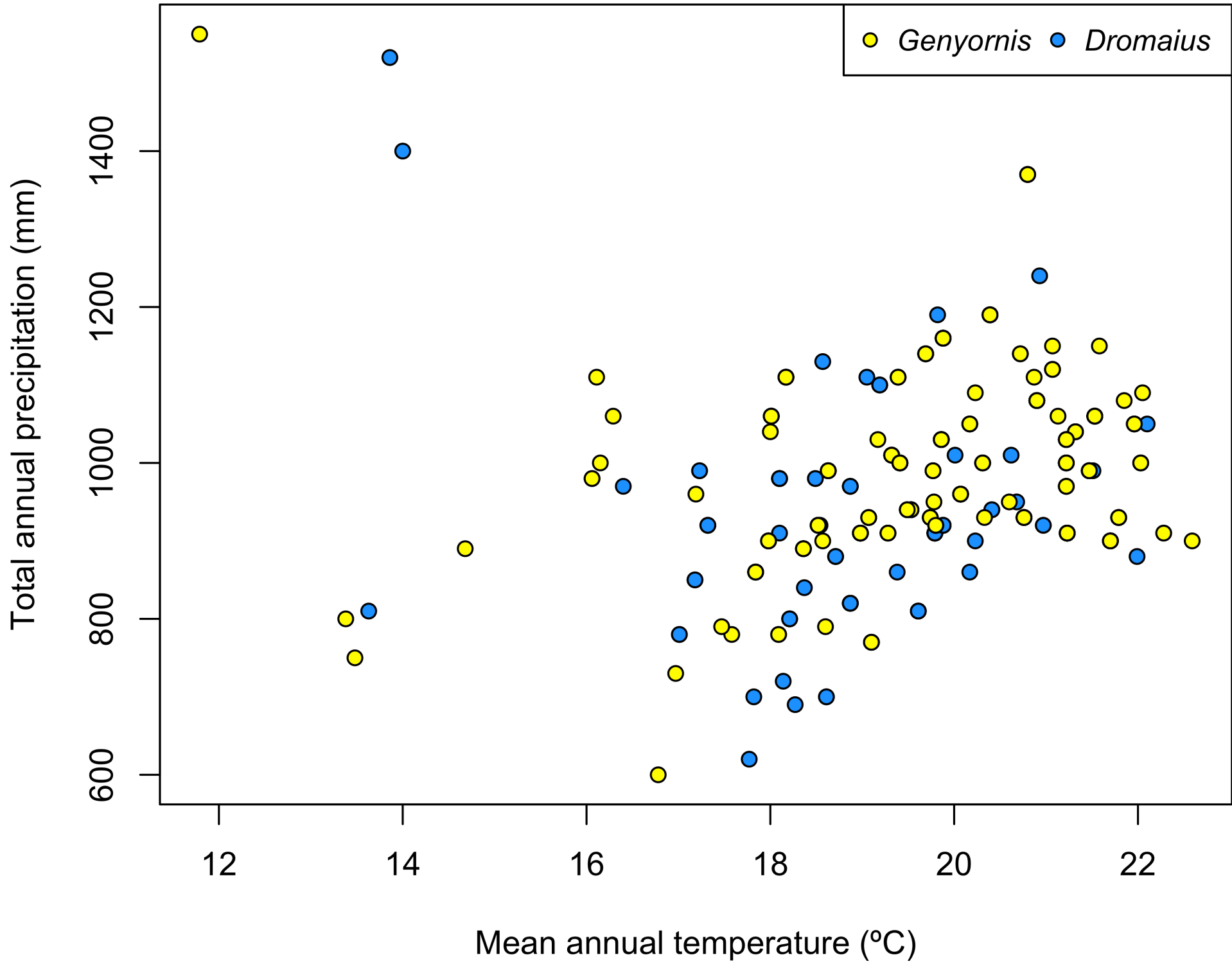

Supplement: S7 Fig — (a) Comparison of climate-envelope dynamics at 56, 46, 34 and 32 ka ago. Circles show fossil locations for each species and darker reds represent higher climatic suitabilities. (b) Overlap of climate-envelopes of both species. (PDF) [file pone.0151090.s008.pdf]
