# Supplementary material for: Where to Dig for Fossils: Combining Climate-Envelope, Taphonomy and Discovery Models
Source: PLoS One. 2016 Mar 30;11(3):e0151090. doi: 10.1371/journal.pone.0151090 (PMC4814095; doi:10.1371/journal.pone.0151090)
Supplement: S1 Table — (PDF) [file pone.0151090.s009.pdf]

S1 Table. Generalised linear models ranked by information criterion.

Model	*k*	*LL*	ΔBIC	*w*BIC	% DE
~ *D1M* + *P*	3	-288.5	0.00	0.207	8.04
~ *D1M* + *P* + *D50K*	4	-285.2	0.07	0.199	9.10
~ *P* + *D50K*	3	-289.2	1.39	0.103	7.82
~ *D1M* + *D50K*	3	-289.4	1.73	0.087	7.76
~ *R* + *D50K*	3	-289.6	2.09	0.073	7.70
~ *D1M* + *P* + *R*	4	-286.3	2.21	0.068	8.76
~ *D1M* + *R*	3	-289.7	2.32	0.065	7.67
~ *P* + *R* + *D50K*	4	-286.4	2.49	0.060	8.72
~ *D1M* + *R* + *D50K*	4	-286.5	2.72	0.053	8.68
~ *D1M*	2	-293.7	3.62	0.034	6.39
~ *D1M* + *P* + *R* + *D50K*	5	-283.6	3.67	0.033	9.60
~ *D50K*	2	-294.4	4.93	0.018	6.18
~ *P* + *R*	3	-294.7	12.36	0.00	6.07
~ *R*	2	-299.5	15.27	0.00	4.53
~ *P*	2	-303.2	22.61	0.00	3.36

Sensitivity analysis examining the relative influence of four sampling-bias related parameters on the presence of fossils. The 15 generalised linear models (logistic regressions) are ranked by their Bayesian information criterion weights (*w*BIC). Also shown are the number of model parameters (*k*), the minimum negative log-likelihood (*LL*), the difference between a model's Bayesian information criterion (BIC) and that of the top-ranked model (ΔBIC), and the percentage of deviance explained (% DE).

Parameters: *D1M* = Distance from centre of a grid cell to the centre of the closest city with > one million inhabitants; *P* = Total population per grid cell; *R* = Total length of roads per grid cell; *D50K* = Distance from center of a grid cell to the centre of the closest city with > 50 thousand inhabitants.
