# Supplementary material for: Where to Dig for Fossils: Combining Climate-Envelope, Taphonomy and Discovery Models
Source: PLoS One. 2016 Mar 30;11(3):e0151090. doi: 10.1371/journal.pone.0151090 (PMC4814095; doi:10.1371/journal.pone.0151090)
Supplement: S2 Table — Estimated coefficients of logistic regression of fossil occurrence as a function of the presence of caves, area of lakes, and area of rocks suitable for fossil preservation. (PDF) [file pone.0151090.s010.pdf]

S2 Table. Summary of fossil preservation model. Estimated coefficients of logistic regression of fossil occurrence as a function of presence of caves, area of lakes, and area of suitable rocks for fossil preservation.

Variable	Estimate	Standard error	Z value	*P* value
(Intercept)	-4.26	1.02	-4.19	< 0.001
Cave presence	1.28	0.25	5.04	< 0.001
Lake area	0.05	0.02	2.40	0.017
Suitable rock area	2.08	1.05	1.99	0.047
