# Supplementary material for: Where to Dig for Fossils: Combining Climate-Envelope, Taphonomy and Discovery Models
Source: PLoS One. 2016 Mar 30;11(3):e0151090. doi: 10.1371/journal.pone.0151090 (PMC4814095; doi:10.1371/journal.pone.0151090)
Supplement: S3 Table — Estimated coefficients of a logistic regression of fossil occurrence as a function of the area of bare soil, mean rain intensity, and mean slope. (PDF) [file pone.0151090.s011.pdf]

S3 Table. Summary of fossil discovery model. Estimated coefficients of logistic regression of fossil occurrence as a function of the area of bare soil, mean rain intensity, and mean slope.

Variable	Estimate	Standard error	Z value	*P* value
(Intercept)	-11.19	1.96	-5.70	< 0.001
Bare soil	0.07	0.02	4.40	< 0.001
Rain intensity*	-19.27	0.03	-7.34	< 0.001
Slope	0.11	0.02	4.63	< 0.001

* Estimated as the mean total annual rainfall divided by the average annual days of rain. Data from the Australian Government's Bureau of Meteorology – www.bom.gov.au
