# Supplementary material for: Where to Dig for Fossils: Combining Climate-Envelope, Taphonomy and Discovery Models
Source: PLoS One. 2016 Mar 30;11(3):e0151090. doi: 10.1371/journal.pone.0151090 (PMC4814095; doi:10.1371/journal.pone.0151090)
Supplement: S4 Table — We compared the suitability values predicted for grid cells containing fossils with unreliable ages of a given genus (not used to train the models) with the suitability values predicted for 1000 sets of randomly selected grid cells from across Australia using a Kolmogorov-Smirnov test. (PDF) [file pone.0151090.s012.pdf]

S4 Table. Validation results for the predictions of the single climate envelope, preservation, and discovery models, as well as their combined predictions. We compared the suitability values predicted for grid cells containing fossils with unreliable ages of a given genus (not used to train the models) with the suitability values predicted for 1000 sets of randomly selected grid cells from across Australia using a Kolmogorov-Smirnov test.

Taxon	Prediction	Median Kolmogorov-Smirnov statistic (1st – 3rd quartiles)
Diprotodon	Palaeo-climate	0.43 (0.36 – 0.57)
	Preservation	0.29 (0.29 – 0.36)
	Discovery	0.57 (0.50 – 0.64)
	Discovery & Preservation	0.50 (0.43 – 0.57)
	All combined	0.50 (0.43 – 0.64)
Zygomaturus	Palaeo-climate	0.67 (0.56 – 0.67)
	Preservation	0.33 (0.22 – 0.33)
	Discovery	0.78 (0.67 – 0.78)
	Discovery & Preservation	0.56 (0.44 – 0.67)
	All combined	0.67 (0.56 – 0.78)
Protemnodon	Palaeo-climate	0.60 (0.53 – 0.67)
	Preservation	0.33 (0.27 – 0.40)
	Discovery	0.40 (0.33 – 0.47)
	Discovery & Preservation	0.53 (0.46 – 0.67)
	All combined	0.60 (0.53 – 0.67)
Thylacoleo	Palaeo-climate	0.75 (0.75 – 0.88)
	Preservation	0.38 (0.25 – 0.38)
	Discovery	0.63 (0.50 – 0.75)
	Discovery & Preservation	0.50 (0.38 – 0.63)

	All combined	0.88 (0.75 – 0.88)
Genyornis	Palaeo-climate	0.63 (0.50 – 0.63)
	Preservation	0.38 (0.38 – 0.50)
	Discovery	0.25 (0.13 – 0.38)
	Discovery & Preservation	0.48 (0.20 – 0.87)
	All combined	0.63 (0.50 – 0.63)
